# Efficacy of a Multi-component m-Health Weight-loss Intervention in Overweight and Obese Adults: A Randomised Controlled Trial

**DOI:** 10.3390/ijerph17176200

**Published:** 2020-08-26

**Authors:** Mitch J. Duncan, Sasha Fenton, Wendy J. Brown, Clare E. Collins, Nicholas Glozier, Gregory S. Kolt, Elizabeth G. Holliday, Philip J. Morgan, Beatrice Murawski, Ronald C. Plotnikoff, Anna T. Rayward, Emmanuel Stamatakis, Corneel Vandelanotte, Tracy L. Burrows

**Affiliations:** 1 School of Medicine & Public Health, Faculty of Health and Medicine, The University of Newcastle, University Drive, Callaghan, NSW 2308, Australia; sasha.fenton@uon.edu.au (S.F.); liz.holliday@newcastle.edu.au (E.G.H.); beatrice.murawski@newcastle.edu.au (B.M.); 2 Priority Research Centre for Physical Activity and Nutrition, The University of Newcastle, University Drive, Callaghan, NSW 2308, Australia; clare.collins@newcastle.edu.au (C.E.C.); philip.morgan@newcastle.edu.au (P.J.M.); ron.plotnikoff@newcastle.edu.au (R.C.P.); anna.rayward@newcastle.edu.au (A.T.R.); tracy.burrows@newcastle.edu.au (T.L.B.); 3 School of Human Movement and Nutrition Sciences, The University of Queensland, St Lucia, QLD 4067, Australia; wbrown@uq.edu.au; 4 School of Health Sciences, Faculty of Health and Medicine, The University of Newcastle, University Drive, Callaghan, NSW 2308, Australia; 5 Brain and Mind Centre, Central Clinical School, The University of Sydney, 94 Mallett St, Camperdown, NSW 2050, Australia; nick.glozier@sydney.edu.au; 6 School of Health Sciences, Western Sydney University, Penrith, NSW 2751, Australia; G.Kolt@westernsydney.edu.au; 7 School of Education, The University of Newcastle, University Drive, Callaghan, NSW 2308, Australia; 8 Charles Perkins Centre, Faculty of Medicine and Health, School of Health Sciences, Sydney 2006, Australia; emmanuel.stamatakis@sydney.edu.au; 9 Physical Activity Research Group, Appleton Institute, School of Health, Medical and Applied Science, Central Queensland University, Rockhampton, QLD 4700, Australia; c.vandelanotte@cqu.edu.au

**Keywords:** adult, diet, m-health, physical activity, sleep, weight loss

## Abstract

Background: This study compared the efficacy of two multi-component m-health interventions with a wait-list control group on body weight (primary outcome), and secondary outcomes of cardiovascular risk factors, lifestyle behaviours, and mental health. Methods: Three-arm randomised controlled trial (Enhanced: physical activity, diet, sleep, Traditional: physical activity, diet, Control) with assessments conducted at baseline, 6 and 12 months. Participants (*n* = 116) were overweight or obese adults aged 19–65 (M = 44.5 [SD = 10.5]). The 6-month intervention was delivered via a smartphone app providing educational materials, goal-setting, self-monitoring and feedback, and also included one face-to-face dietary consultation, a Fitbit and scales. The trial was prospectively registered and conducted between May 2017 and September 2018. Group differences on primary and secondary outcomes were examined between the Pooled Intervention groups (Pooled Intervention = Enhanced and Traditional) and Control groups, and then between Enhanced and Traditional groups. Results: Nineteen participants (16.4%) formally withdrew from the trial. Compared with the Control group, average body weight of the Pooled Intervention group did not differ at 6 (between-group difference = −0.92, (95% CI −3.33, 1.48)) or 12 months (0.00, (95% CI −2.62, 2.62)). Compared with the Control group, the Pooled Intervention group significantly increased resistance training (OR = 7.83, (95% CI 1.08, 56.63)) and reduced energy intake at 6 months (−1037.03, (−2028.84, −45.22)), and improved insomnia symptoms at 12 months (−2.59, (−4.79, −0.39)). Compared with the Traditional group, the Enhanced group had increased waist circumferences (2.69, (0.20, 5.18)) and sedentary time at 6 months (105.66, (30.83, 180.48)), and improved bed time variability at 12 months (−1.08, (−1.86, −0.29)). No other significant differences were observed between groups. Conclusions: Relative to Controls, the Pooled Intervention groups did not differ on body weight but improved resistance training, and reduced energy intake and insomnia symptom severity. No additional weight loss was apparent when targeting improvements in physical activity, diet and sleep in combination compared with physical activity and diet.

## 1. Introduction

Globally, overweight and obesity are significant contributors to morbidity and mortality [1]. Traditionally, behavioural weight loss interventions aim to increase physical activity, improve diet quality and reduce total energy intake to promote weight loss [2]. Although traditional weight loss interventions achieve modest short-term weight loss (e.g., <3.80 kg over 6 months) relative to control groups [3,4], improving sleep in combination with physical activity and diet has the potential to contribute to greater weight loss than interventions only targeting physical activity and diet [5,6,7]. This is relevant as between 20 and 30% of adults engage in a pattern of behaviours characterised by low levels of physical activity, poor dietary behaviours and insufficient sleep [8].

Optimal sleep health is characterised by duration, quality and timing of sleep that leaves a person satisfied with their sleep and alert during the day [9]. Potential mechanisms linking sleep with weight regulation include reduced activity levels among people with short duration or poor quality sleep [10,11]. Shorter sleep duration (<5.5 hrs/night) is also associated with increased energy intake, greater likelihood to select energy-dense foods, and poorer regulation of hunger [12,13,14]. Additionally, having poorer quality, shorter sleep duration (<7 hrs/night), or sleep-disordered breathing at the start of a traditional weight loss intervention is associated with lower weight loss in intervention studies [15,16]. However, these studies did not specifically aim to improve sleep as part of the intervention [15,16]. To our knowledge, only one study has examined how improving sleep, in combination with physical activity and diet, influenced weight loss in adults [5]. This study reported greater weight loss from a physical activity, diet and sleep health intervention (5%) compared to an intervention without a sleep component (2%) [5]. The sleep component of the combined intervention commenced in week 4 of the 12-week intervention, potentially limiting the improvements in sleep and reducing potential impacts on weight loss [5]. Despite these promising results, further research is necessary to examine how improving sleep influences weight loss over longer periods of time.

Due to the high prevalence of overweight and obesity, it is important that intervention approaches have the potential for wide reach and access [3,17]. Interventions which include electronic components such as e-health (i.e., website) and m-health (i.e., apps) offer opportunities for delivering behavioural and weight loss interventions in a format that is scalable, and allow participation at times that are convenient to individuals [18,19]. Few (*n* = 4) m-health interventions target physical activity, diet and sleep behaviours in combination, and none have focussed on weight loss [20]. The primary aim of the current study was to compare the efficacy of two multi-component m-health interventions pooled together with a wait-list Control group on body weight (primary outcome) and secondary outcomes including cardiovascular risk factors (i.e., waist circumference, HbA1c), behaviours (i.e., physical activity, diet, sleep) and mental health (i.e., depression, anxiety and stress symptoms). The secondary aim was to compare the relative efficacy of a physical activity, dietary behaviour and sleep intervention (Enhanced intervention), with a physical activity and dietary behaviour only intervention (Traditional intervention) on both primary and secondary outcomes. The hypotheses were that the both the Enhanced and the Traditional interventions would achieve greater weight loss than the Control group, and that the Enhanced intervention would achieve greater weight loss than the Traditional intervention.

## 2. Methods

### 2.1. Trial Design

**Figure 1:**
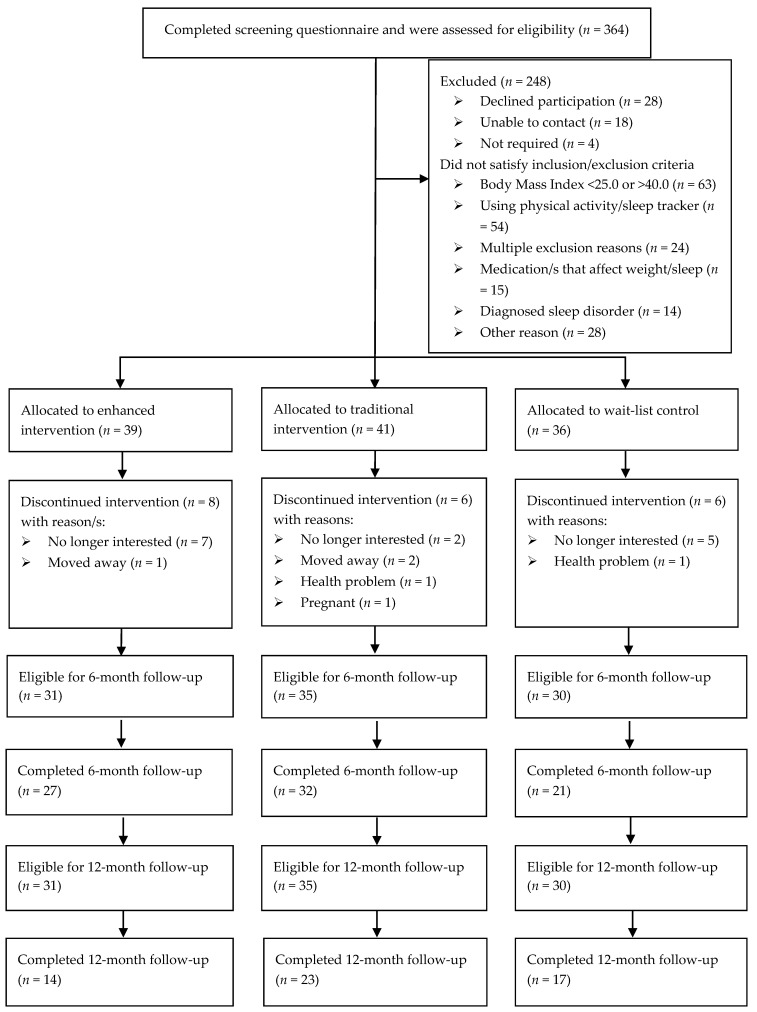
Move, Eat and Sleep Study flow chart.

The Move, Eat and Sleep study was a three-arm randomised controlled trial (RCT) with in-person assessments conducted at baseline, 6 months (primary end point) and 12 months (Figure 1). The detailed study protocol has been published earlier [6]. In summary, adults (*n* = 116; Female = 82) living in the Newcastle area, New South Wales, Australia were recruited (May–September 2017), primarily by newspaper and radio stories, participant registries, and electronic communication (email lists, social media advertising). Inclusion criteria were age 18–65 years, a BMI between 25.0 and 40.0 kg/m^2^, and possession of an iOS/Android smartphone/tablet with internet access. Exclusion criteria were current use of an activity tracker for physical activity and/or sleep, current pregnancy, reported presence of a doctor-diagnosed sleep disorder, current use of medication to assist with sleep or weight management, presence of a condition which precluded activity, diet and/or sleep behaviour modification, weight loss ≥4.5 kg in last 3 months, intention to participate in another weight loss trial, previous weight loss surgery at any time, or current employment involving shift-work on a rotating roster.

All assessments were completed at the University of Newcastle. After completing an online eligibility survey, individuals were contacted by project staff to complete visit 1 where eligibility status was confirmed. At visit 1, eligible participants had their HbA1c, height and weight measured, completed the Australian Eating Survey and were provided with an accelerometer (Geneactiv) to wear continuously for 8 days. At visit 2 (9 days later), participants returned the accelerometer and completed an online survey.

After completing baseline assessments at visit 2, participants were randomly allocated (1:1 ratio) to one of the three study groups using secure, web-based randomisation. Allocation was stratified by baseline BMI (25.0–29.9; 30.0–40.0) using block sizes of 6. Participants who shared their residence with another participant were allocated to the same group to avoid contamination between groups. The research assistants who conducted the anthropometric assessments were blinded to group allocation. The dietitians who provided dietary counselling and participants were not blinded to group allocation.

Follow-up assessments were conducted at 6 and 12 months (November 2017–September 2018) post-randomisation. Participants were mailed the accelerometer prior to the 6- and 12-month assessments and received a gift voucher (AUD 10) at the completion of each assessment. The study was approved by the Human Research Ethics Committee of the University of Newcastle Australia (Reference number H-2017-0039) and was prospectively registered (ACTRN12617000735358; Universal Trial Number U1111-1219-2050). All participants provided informed consent to participate. The funding bodies had no role in the design, conduct or reporting of the trial.

#### 2.1.1. Intervention

The Enhanced and Traditional group participants were provided with personalised dietary recommendations, and given access to the ‘Balanced’ smartphone app [6], an additional calorie counting platform (CalorieKing Wellness Solutions Inc,, La Mesa, CA, USA), a set of body weight scales (Tanita HD-380), a Fitbit activity tracker (Fitbit Alta) and a participant handbook [6,21,22]. The intervention used behaviour change techniques (e.g., education, goal setting, self-monitoring, feedback on behaviour) to operationalise constructs from social cognitive and self-regulatory theories specifical to the target behaviours [23,24,25]. An overview of the intervention is provided in Supplementary Table S1. Participants received intervention content specific to their group allocation only.

Educational materials detailing how behaviours related to weight loss and target weight loss (5% weight loss target) were provided via in-app content, email and SMS messages, a printed participant handbook, and in-person via a dietary counselling session. The handbook provided guidance on goal setting, action planning, stress management, healthy eating advice and body weight resistance training activities. The in-person counselling session followed a standardised protocol to provide personalised dietary advice based on assessment of their current dietary intake, as measured by the Australian Eating Survey^®^ (FFQ) and personalised nutrition report (Australian Eating Survey^®^ Version 2, University of Newcastle, Callaghan NSW, Australia) to improve overall diet quality in line with Australian Dietary Guidelines and the Australian Guide to Healthy Eating [26,27,28,29]. Participants were also provided with a personalised daily energy intake target to create an energy deficit of 2000 kJ based on the Mifflin-St Jeor equation [30]. Intervention group participants accessed the Balanced app to set goals and self-monitor weight and target behaviours (daily minutes of moderate-to-vigorous intensity physical activity, steps, resistance training, and the number of food goals achieved), and received dynamic feedback on performance relative to their goals [6]. Both intervention groups were also encouraged to self-monitor and receive feedback on overall diet quality [31] and energy intake (kilojoules/day) (CalorieKing website or the ControlMyWeight app by CalorieKing) [6].

The Enhanced group also received access to a sleep intervention via the app and participant handbook [6,21,22] that targeted a reduction in sleep timing variability, promoted sleep hygiene behaviours and provided stress management and relaxation techniques (e.g., progressive muscle relaxation, deep breathing exercises, and mindfulness). The Enhanced group could set goals and self-monitor and receive dynamic feedback on bed times/wake times, sleep quality and sleep hygiene behaviours [6,21,22]. Participants of both intervention groups received emailed weekly summaries of their behaviours in relation to their goals based on Balanced app entries, and weekly educational weight loss facts via SMS. Participants who stopped self-monitoring using the app (≥4/7 days/week) were sent an SMS to prompt re-engagement [6,21,22].

#### 2.1.2. Measures

Body weight (kg, primary outcome) and height (cm) were measured on calibrated digital scales (Biospace BSM370 Portable Automatic BMI Stadiometer, Biospace Co, Ltd., Seoul, Korea). Weight was measured twice in light clothing and without shoes, measures were considered consistent if they differed by <0.1 kg; if measures varied by more than 0.1 kg a third measurement was taken. Waist circumference (centimetres) was measured at the narrowest point between the lower costal border and iliac crest (Seca 203, Seca Gmph & Co., Hamburg, Germany). Waist circumference was measured twice in light clothing, measures were considered consistent if they differed by <0.5 cm, if measures varied by more than 0.5 cm a third measurement was taken. Using a capillary blood sample, HbA1c was measured using capillary blood sample (A1C Now+ (Polymer Technology Systems)).

Participants were asked to wear an accelerometer (Geneactiv) on their non-dominant wrist for 8 consecutive days, 24 hrs per day, and completed a monitoring log of bed/wake times and periods of non-wear [6]. Valid wear time was defined as at least 16 hrs of wear time per day on at least five days [6,32]. Raw accelerometer data were processed using GGIR (version 1.10-1) in R and incorporated participant monitoring logs to estimate the average number of nightly awakenings as an indicator of sleep quality, time spent in inactivity as an indicator of sedentary behaviour, light (50–100 milligravitational units (mg)) and moderate-to-vigorous intensity physical activity (>100 mg) [32,33,34,35,36].

The psychometric properties of the instruments used to assess secondary outcomes are detailed elsewhere [6]. Secondary outcomes included weekly minutes of moderate- and vigorous-intensity physical activity (as measured by the Active Australia Survey) [37,38,39], weekly frequency of resistance training [6,21,22], daily sitting time (Workforce Sitting Questionnaire) [40], daily energy intake and dietary quality (Australian Eating Survey) [26,27], sleep quality (Pittsburgh Sleep Quality Index (PSQI) [41], insomnia symptom severity (Insomnia Severity Index (ISI)) [42], sleep timing (Sleep Timing Questionnaire) [43,44]. Participant satisfaction with and usability of the Balanced app was assessed using the System Usability Scale [45]. The Balanced app database tracked any self-monitoring entries made by participants and was used to determine the time to non-usage attrition (defined as the first occurrence of a 14 consecutive day period of no self-monitoring) [21,22]. Online surveys were used to collect participant characteristics (i.e., age, sex, marital status, ethnicity, medical history, postal code, years of education, occupational level, hours of work (daytime, night time, afternoon), number of days worked in previous week, average hours of work each day, diurnal preference) [46,47].

This trial was powered to detect a between-group difference (Pooled Intervention groups vs. Control) in the primary outcome of body weight at the primary endpoint of 6 months post-randomisation. Assuming a mean between-group difference in body weight of 3.6 kg, standard deviation of 13.4 kg, and a pre-post within-person correlation of 0.9, 31 participants per group were required to provide 80% power at 0.05 significance [6]. A total 38 participants per group were recruited to allow for an anticipated ~17% attrition (total *n* = 116) [21].

### 2.2. Statistical Analyses

Analyses followed an intention-to-treat principle using all available data. Characteristics of completers and non-completers (i.e., non-completion of the 6-month assessment) were compared using t-tests and chi-squared tests. The proportions of participants reporting an acceptable system usability score (≥70) [45], mean system usability scores and self-monitoring entries were compared between intervention groups using chi-squared tests and t-test. Two series of analyses were conducted to compare between-group differences in primary and secondary outcomes. The first compared the Pooled Intervention groups (Enhanced and Traditional groups) and the Control group on primary and secondary outcomes. The second set of analyses compared the Enhanced and Traditional groups on primary and secondary outcomes. Between-group differences in primary and secondary outcomes at 6 and 12 months were examined using Generalized Linear Mixed Models (GLMM) using an ANCOVA (baseline-adjusted) approach. The GLMMs included fixed effects for the baseline value of the outcome, group, time, the group × time interaction, the BMI stratification variable, a random intercept to account for repeated measures of participants and accounted for participant clustering in households. The distribution of response data and residual diagnostics informed the selection of family and link functions. The magnitude of the differences between groups using observed values at 6 and 12 months calculated was also expressed as Cohen’s d (95% CI). Between-group differences in the time to non-usage attrition were made using Cox proportion hazards regression and plotted using Kaplan–Meier survival estimates. Multiple imputation was performed for sensitivity analyses, with ten datasets being imputed, including variables predicting missingness or the outcome. Data were imputed using Chained Equations and Predicted Mean Matching. Analyses were conducted using Stata MP (15.1) in October 2019. An alpha level of 0.05 was used for the primary outcome and all secondary outcomes.

## 3. Results

Of the 116 participants who completed baseline assessments, 80 (69.8%) completed the 6-month assessment and 54 (46.6%) completed the 12-month assessment (Figure 1). The completion rates did not differ between groups (*p* = 0.193). The reasons for formal withdrawal from the trial are detailed in Figure 1. The overall dropout rate (defined as formal withdrawal from the trial) was 16.4% (*n* = 19) and was similar across groups. Completers and non-completers were similar for sociodemographic, weight, and behavioural characteristics, except completers reported fewer symptoms of depression and stress (Supplementary Table S2). A total of 6 household dyads (Control: 1; Traditional: 2; Enhanced: 3) and one triad were included (Traditional: 1). Participant baseline characteristics by study group, are shown in Table 1. The mean participant age was 44.5 years (range 19.8–64.5), 70% were female and approximately half were employed fulltime. The mean weight was 90.7 kg and 70% were classified as obese.

### 3.1. Weight and Behavioural Outcomes

**Figure 2:**
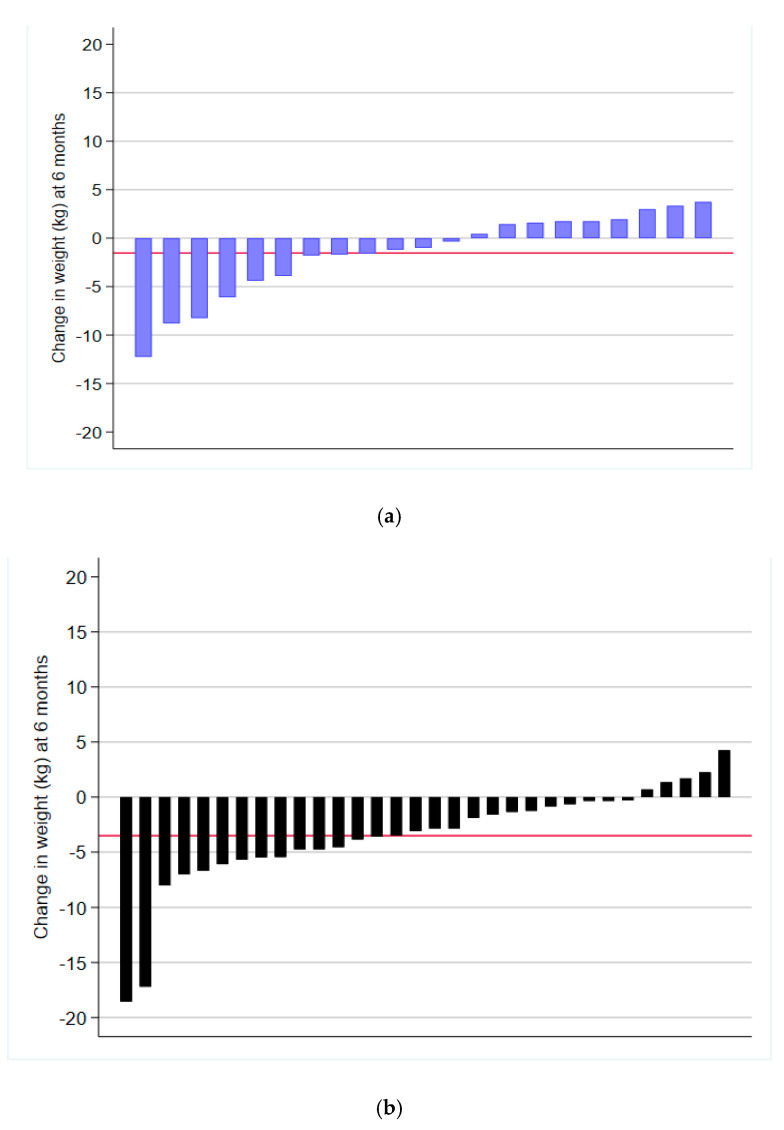

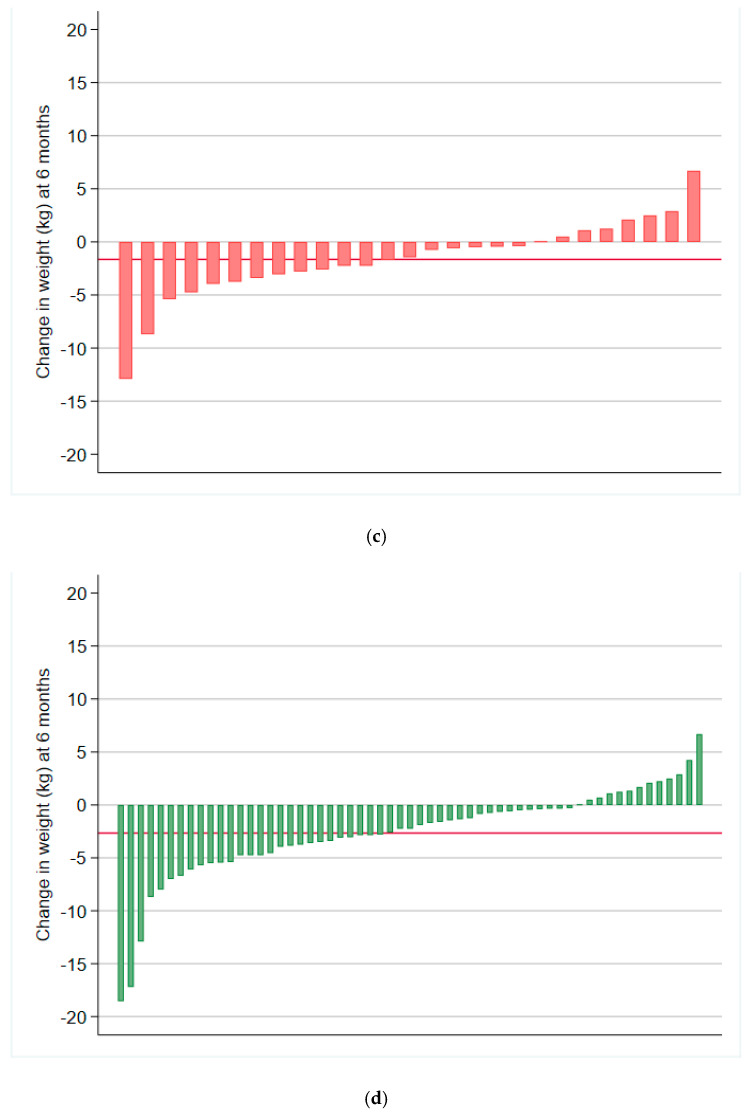
Individual weight change from baseline to 6 months by study group. (**a**) Control group participant change at 6 months; (**b**) Traditional group participant change at 6 months; (**c**) Enhanced group participant change at 6 months; (**d**) Pooled group participant change at 6 months.

**Figure 3:**
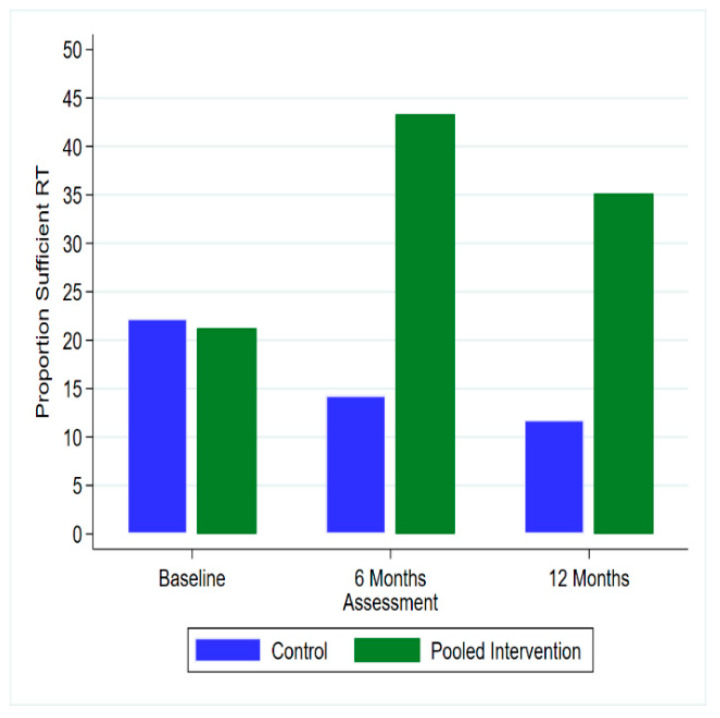
Proportion of Control Group and Pooled participants reporting sufficient resistance training (RT) at Baseline, 6 months and 12 months.

At 6 months, weight was not significantly different between the Pooled Intervention groups and Control group (difference = −0.92, 95% CI (−3.33, 1.48)) or 12 months (difference = 0.00, 95% CI (−2.62, 2.62)) (Table 2). Individual weight change and the group mean weight change from baseline to 6 months and 12 months is shown in Figure 2a–d and Supplementary Figure S1a–d, respectively. Daily energy intake was significantly lower in the Pooled Intervention group than the Control group at 6 months (difference = −1037.03, 95% CI (−2028.84, −45.22)) but not at 12 months (difference = −913.36, 95% CI (−2030.75, 204.04)) (Table 2). Severity of insomnia symptoms was significantly lower in the Pooled Intervention group than the Control group at 12 months (difference = −2.59, 95% CI (−4.79, −0.39)) (Table 2). The Pooled Intervention group was significantly more likely to report meeting resistance training guidelines than the Control group at 6 months (OR = 7.83, 95% CI (1.08, 56.63), *p* = 0.041), but not at 12 months (OR = 5.68, 95% CI (0.55, 58.83), *p* = 0.145) (Figure 3). There were no other significant differences between the Pooled Intervention group and the Control group at either 6 or 12 months (Table 2).

Figure 2 Notes. Only includes participants with weight measured at 6 months. Average (SD) weight loss at 6 months: Control (*n* = 21) = −1.54 (4.35); Traditional (*n* = 32) = −3.51 (4.77); Enhanced (*n* = 27) = −1.65 (3.77); Pooled (*n* = 59) = −2.66 (4.40). Red line indicates average weight loss for group.

Figure 3 Notes. Only includes participants with weight measured at 6 months. The Pooled Intervention group was significantly more likely to report meeting resistance training guidelines than the Control group at 6 months (OR = 7.83, 95% CI (1.08, 56.63), *p* = 0.041), but not at 12 months (OR = 5.68, 95% CI (0.55, 58.83), *p* = 0.145). Data are available from observations; the proportion reporting sufficient RT (2+ Days/week) in the Control group was 8/36 (22.2%), 3/21 (14.3%) and 2/17 (11.8%) at baseline, 6 months and 12 months; for the Pooled Intervention group this was 17/80 (21.3%), 26/60 43.3%) and 13/37 (35.1%) at baseline, 6 months and 12 months, respectively.

When comparing the two intervention groups, waist circumference was significantly lower in the Traditional group compared to the Enhanced group at 6 months (difference = 2.69, 95% CI (0.20, 5.18)), but not at 12 months (difference = 0.75, 95% CI (−2.08, 3.59)) (Table 3). Sedentary time was significantly lower in the Traditional group than in the Enhanced group at 12 months (difference =105.66, 95% CI (30.83, 180.48)) (Table 3). Bed time variability was significantly lower (improved) in the Enhanced group at 12 months (difference = −1.08, 95% CI (−1.86, −0.29)) (Table 3). There were no other differences between the two intervention groups at either 6 or 12 months. Supplementary Tables S3 and S4 present the descriptive statistics for the primary and secondary outcomes and indicate that the magnitude of differences between groups (Cohen’s d) at each assessment point were of a small-to-moderate magnitude at 6 months and were smaller at 12 months. Sensitivity analyses using multiple imputation for missing data identified a similar pattern of results (Supplementary Tables S5 and S6).

### 3.2. Usage Outcomes

**Figure 4:**
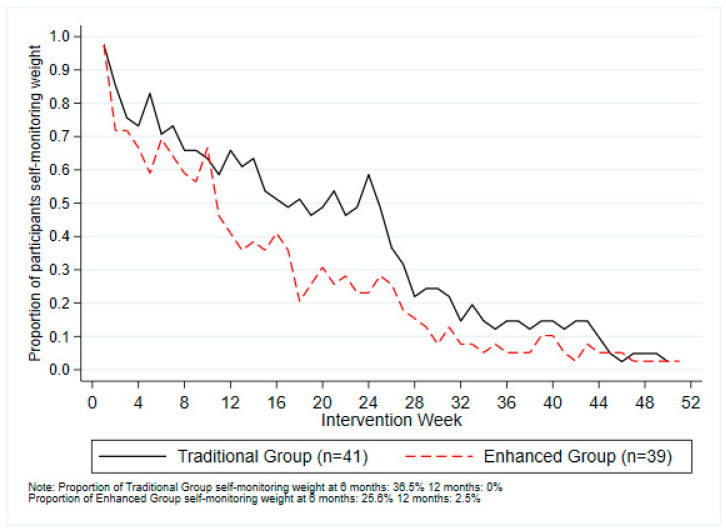
Frequency of weekly self-monitoring of body weight using the Balanced app by intervention participants.

**Figure 5:**
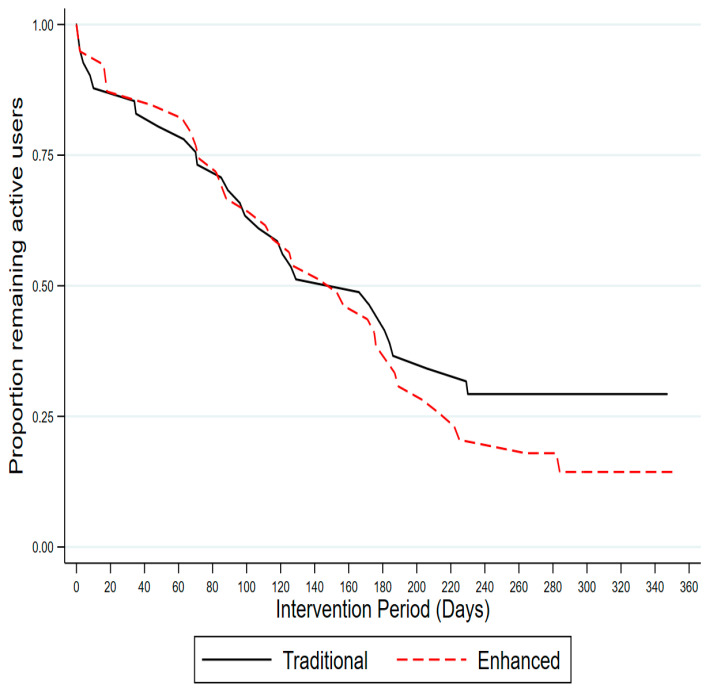
Kaplan–Meier estimates of the survival distribution for time to non-usage attrition by group.

Over the 12-month study period, the mean total number of self-monitoring entries did not differ between the two intervention groups (Traditional = 156.5 (±102.8); Enhanced = 140.4 (±83.3), *p* = 0.440) (Table 4). The average number of self-monitoring entries was lower for participant-entered metrics (e.g., Food, Body Weight) than for the metrics automatically entered by the Fitbit (e.g., Fitbit MVPA) (Table 4). The proportion of intervention group participants who self-monitored weight gradually declined during the intervention (Figure 4). Over the 12-month period, a total of 62 (77.5%) participants succumbed to non-usage attrition (Traditional = 29 (70.7%); Enhanced = 33 (84.6%)), and there was no statistically significant difference between intervention groups (HR = 1.26, 95% CI 0.77, 2.08, *p* = 0.360) (Figure 5). The overall mean system usability score (SUS) was 71.5 (±17.3), and did not significantly differ between the Traditional (74.0 (±14.2)) and Enhanced groups (68.5 (±20.6)), *p* = 0.109). The proportion of responses to the individual SUS items by intervention group is shown in Supplementary Table S7.

## 4. Discussion

The primary aim of the current study was to compare the effect of the Pooled Intervention compared to the Control group on the primary outcome of body weight. Body weight did not significantly differ between the Pooled Intervention group compared to the Control at 6 or 12 months, indicating weight loss did not differ between the groups. The Pooled Intervention group reduced energy intake, insomnia symptom severity, and increased resistance training in comparison to the Control group. While body weight did not significantly differ between the two interventions groups, the Traditional group improved waist circumference and sedentary time in comparison to the Enhanced group, and the Enhanced group improved (reduced) the variability in bed time in comparison the Traditional group.

Findings from meta-analyses have shown that e- and m-health weight loss interventions are effective in comparison to control groups [3,20]. However, in the current study, body weight did not significantly differ between any of the study groups and this may be due to several potential reasons. Importantly, all groups lost weight relative to baseline including the Control group (Figure 2a–d). Although weight loss among control group participants is commonly reported in behavioural weight loss interventions [48], the magnitude of weight loss in the Control group (−1.54 kg at 6 months) in the current study was not anticipated. Potential explanations for this include participants volunteering to participate in a weight loss trial, being allocated to the wait list Control group and engaging in alternative weight loss strategies despite their group allocation. Although no information is available concerning if Control group participants did participate in other weight loss interventions, all participants were asked to not participate in other weight loss interventions during the study.

The physical activity intervention used in this study has been shown to increase resistance training in previous studies [21,22], this is consistent with the greater frequency of resistance training in the Pooled Intervention relative to the Control group observed in the current study. Participation in resistance training has important health benefits [4,49]. However, it is unclear how the greater frequency of resistance training in the Pooled Intervention influenced the lean muscle mass, body composition and corresponding body weight of these participants and offset any differences in weight loss relative to the Control group. Examining body composition and related outcomes would help to clarify this issue in future studies. Additionally, inclusion criteria did not consider participant lifestyle behaviours, and participants had relatively high levels of moderate-to-vigorous intensity physical activity, and sub-clinical levels of insomnia at baseline [42]. Consequently, despite modest reductions in energy intake at 6 months, participants may have had limited ability to further improve target behaviours and achieve weight loss over the study period.

With the exception of reduced (i.e., improved) bed time variability in the Enhanced group, no other sleep outcome differed between the two intervention groups. Though insomnia treatments typically focus on reducing wake time variability to help improve sleep drive [50], reduced bed time variability is part of sleep hygiene recommendations (e.g., have a regular sleep pattern), and is associated with less frequent insufficient sleep [44]. The reduced bed time variability may reflect changes in participant behaviours to improve sleep in the current study. In trials that included participants with poorer sleep quality and more severe insomnia symptoms than in the current trial, the sleep interventions improved various indicators of sleep health at three and 6 months [21,22]. As cognitive and/or behavioural interventions for sleep produce larger improvements when participants have poorer sleep quality at baseline [51], participants in the current study may have had limited the potential for improvement in sleep quality. Additionally, both intervention groups received the same physical activity intervention and were more likely to meet resistance training guidelines at 6 months than the Control group. As resistance training has been shown to improve sleep quality [52] this may have attenuated the additional effect of the sleep intervention in the Enhanced group [22].

Although the mechanisms are not well established [7], poor sleep may contribute to weight gain by increased energy intake [12,17] and reduced physical activity levels [11,53]. Consequently, it was hypothesised that the Enhanced group which included the sleep intervention would produce greater weight loss and improvements in activity and diet behaviours than the Traditional group; this did not occur. Most studies that have examined how sleep influences diet are short term (<5 days) laboratory-based experiments that restrict the sleep duration (<5.5 hrs/d) of people with recommended sleep durations (i.e., 7–9 hrs/d) [12,13,54]. In addition, the previously mentioned study that reported greater weight loss when physical activity, diet and sleep were targeted, compared to physical activity and diet, only examined weight loss over 12 weeks [5]. By comparison, the current study included participants with mostly sub-clinical levels of insomnia and average sleep durations of 6.5 hrs/d, and aimed to improve sleep over six months in the community setting in the context of a weight loss intervention. These differences may partly explain why the two intervention groups did not differ on weight and energy intake. Moreover, as the dose–response relationship between improved sleep (i.e., dimension of sleep health, magnitude and duration of change) and daily energy intake is unclear, it is possible that the modest improvements in sleep quality and insomnia symptoms over the study period were not sufficient to have any influence on total energy intake.

Self-monitoring behaviours and weight are important determinants of behaviour change and weight loss, respectively [3,55]. While there is no consensus on the amount of use and engagement needed to change behaviours, consistent with the current study, most m-health and e-health interventions report that usage declines over time [21,22,55,56,57]. The proportion of participants who succumbed to non-usage attrition in the current study (Traditional = 70.7%; Enhanced 84.6%) did not differ between intervention groups and is broadly comparable to other interventions using the Balanced app to target physical activity and sleep combination (68–89%) [21,22]. However, direct comparisons between studies are difficult due to differences in self-monitoring methods as the current study used a combination of Fitbit and manual entry, and the previous studies only used manual entry [21,22]. As the Enhanced group had a significantly lower number of self-monitoring entries that were manually entered by participants (i.e., steps, resistance training, food, and weight) (Table 4), it is possible that the self-monitoring requirements in the Enhanced group were too burdensome. This issue combined with the number of different intervention components and delivery modalities used in the intervention (see Table S1) may have overwhelmed some participants limiting their behavioural changes and subsequent weight loss [58]. Although efficacy of simultaneous and sequentially delivered interventions does not appear to differ [58] the self-monitoring differences between groups highlights the importance of carefully targeting and operationalising intervention components in simultaneous multiple behaviour interventions [59,60].

The current study has several strengths. These include the randomised design, the length of the intervention and follow-up relative to many weight loss interventions, and targeting improved sleep health as part of a weight loss intervention [3,54]. Limitations, however, include the potential presence of undiagnosed sleep conditions (although the baseline scores on the PSQI and insomnia severity index indicate participants did not have severely impaired sleep). Further, the wrist worn accelerometer is less useful than thigh worn devices for assessing sedentary behaviour, due to the postural component needed to accurately define sedentary behaviour [61].

In conclusion, relative to the Control group, the Pooled Intervention group did not differ in terms of body weight, but improved resistance training, and reduced energy intake and insomnia symptom severity. Additional improvements in weight loss, physical activity, diet and sleep behaviours associated with targeting improvements in physical activity, diet and sleep in combination compared to physical activity and diet were not evident. Participants had relatively favourable behavioural profiles at baseline which may have limited the ability of participants to further improve behaviours, and all groups lost weight relative to baseline potentially limiting the ability to detect between-group differences in weight loss.

## Figures and Tables

**Table 1:** Baseline sociodemographic, health and behavioural characteristics of participants by group.

	Pooled Intervention	Enhanced	Traditional	Control	Total
	*N* = 80	*N* = 39	*N* = 41	*N* = 36	*N* = 116
	M (SD), N (%)	M (SD), N (%)	M (SD), N (%)	M (SD), N (%)	M (SD), N (%)
Age (years)	46.3 (9.8)	47.2 (9.4)	45.4 (10.2)	40.5 (10.7)	44.5 (10.4)
Sex					
Female	57 (71.3%)	27 (69.2%)	30 (73.2%)	25 (69.4%)	82 (70.7%)
Male	23 (28.7%)	12 (30.8%)	11 (26.8%)	11 (30.6%)	34 (29.3%)
Years Education	17.1 (2.9)	17.3 (3.0)	16.9 (2.7)	14.8 (2.7)	16.4 (3.0)
Marital Status					
Married/Defacto	63 (78.8%)	30 (76.9%)	33 (80.5%)	29 (80.6%)	92 (79.3%)
Not Married	17 (21.3%)	9 (23.1%)	8 (19.5%)	7 (19.4%)	24 (20.7%)
Employment					
Full time	43 (53.8%)	21 (53.8%)	22 (53.7%)	19 (52.8%)	62 (53.4%)
Part time/casual	26 (32.5%)	11 (28.2%)	15 (36.6%)	13 (36.1%)	39 (33.6%)
Retired	6 (7.5%)	3 (7.7%)	3 (7.3%)	0 (0.0%)	6 (5.2%)
Other	5 (6.3%)	4 (10.3%)	1 (2.4%)	4 (11.1%)	9 (7.8%)
Weight (kg)	89.8 (13.4)	90.8 (13.1)	88.9 (13.8)	92.5 (16.1)	90.7 (14.3)
Measured BMI	31.7 (3.9)	31.9 (4.0)	31.4 (3.8)	31.9 (3.9)	31.7 (3.9)
Measured BMI Categories					
BMI 25.0–29.9	25 (31.3%)	11 (28.2%)	14 (34.1%)	10 (27.8%)	35 (30.2%)
BMI 30.0–40.0	55 (68.8%)	28 (71.8%)	27 (65.9%)	26 (72.2%)	81 (69.8%)
Waist Circ. (cm)	99.6 (10.8)	99.5 (12.5)	99.6 (9.0)	99.7 (11.7)	99.6 (11.0)
HbA1c	5.5 (0.6)	5.5 (0.4)	5.5 (0.7)	5.3 (0.3)	5.4 (0.5)
Self-reported MVPA (mins.wk)	346.4 (316.3)	341.5 (270.6)	351.0 (357.7)	238.1 (239.2)	312.8 (297.8)
Resistance Training Freq. (d.wk)					
0–1 Session	63 (78.8%)	32 (82.1%)	31 (75.6%)	28 (77.8%)	91 (78.4%)
2+ Session	17 (21.3%)	7 (17.9%)	10 (24.4%)	8 (22.2%)	25 (21.6%)
MVPA (mins.d) ‡	56.4 (33.3)	56.6 (34.0)	56.3 (33.0)	49.2 (24.2)	54.4 (31.1)
Light Intensity Activity (mins.d) ‡	197.8 (76.4)	199.8 (74.3)	196.1 (79.1)	188.0 (66.0)	195.1 (73.5)
Daily Sitting Time (mins.d)	703.0 (205.5)	667.4 (194.5)	736.8 (212.2)	678.1 (183.6)	695.3 (198.5)
Sedentary Time (mins.d) ‡	617.7 (121.7)	606.0 (143.5)	629.7 (94.8)	584.0 (180.7)	607.4 (142.3)
Energy Intake (kj.d)	9921.6 (3274.0)	9422.1 (3518.6)	10,396.9 (2989.0)	9152.6 (2810.3)	9683.0 (3145.6)
Total diet quality (ARFS 0–73)	36.3 (8.4)	36.5 (9.0)	36.0 (8.0)	33.3 (10.0)	35.4 (9.0)
PSQI Global Score	7.1 (3.0)	7.3 (2.8)	7.0 (3.1)	6.7 (3.0)	7.0 (3.0)
PSQI Sleep Quality Classification					
Good Quality Sleep	18 (22.5%)	7 (17.9%)	11 (26.8%)	8 (22.2%)	26 (22.4%)
Poor Quality Sleep	62 (77.5%)	32 (82.1%)	30 (73.2%)	28 (77.8%)	90 (77.6%)
Insomnia Severity Index	9.2 (4.9)	9.9 (4.6)	8.5 (5.2)	7.6 (4.8)	8.7 (4.9)
PSQI Sleep Duration (hrs.d)	6.8 (1.0)	6.7 (1.1)	6.8 (1.0)	6.8 (0.9)	6.8 (1.0)
Sleep Duration (hrs.d) ‡	6.4 (0.9)	6.4 (0.9)	6.4 (0.9)	6.7 (0.7)	6.5 (0.9)
Bed time Variability	3.4 (1.7)	3.5 (1.8)	3.2 (1.6)	3.0 (1.4)	3.3 (1.6)
Wake time Variability	2.4 (1.3)	2.3 (1.3)	2.6 (1.4)	2.4 (1.3)	2.4 (1.3)
Nightly Awakenings ‡	2.6 (1.6)	2.8 (1.8)	2.3 (1.3)	1.6 (1.4)	2.3 (1.6)
DASS-Depression	6.2 (6.3)	5.8 (4.9)	6.5 (7.4)	8.4 (7.9)	6.9 (6.9)
DASS-Anxiety	4.5 (4.9)	4.2 (4.5)	4.7 (5.2)	5.1 (5.8)	4.7 (5.2)
DASS-Stress	10.8 (6.4)	10.8 (6.1)	10.8 (6.8)	10.9 (6.8)	10.8 (6.5)

Notes. ‡ Measured using accelerometer.

**Table 2:** Baseline adjusted group differences between Pooled and Control groups at 6 months and 12 months.

Outcome	Control	Pooled Intervention	Group Difference (95% CI)
	M (95% CI)	M (95% CI)	
**Weight (kg)**			
6 Months	87.67 (85.58, 89.75)	86.75 (85.45, 88.04)	−0.92 (−3.33, 1.48)
12 Months	87.21 (85.00, 89.43)	87.21 (85.74, 88.69)	−0.00 (−2.62, 2.62)
**Waist Circ. (cm) ‡,†**			
6 Months	97.14 (94.91, 99.37)	96.52 (95.14, 97.90)	−0.62 (−3.18, 1.95)
12 Months	95.45 (93.10, 97.80)	96.04 (94.49, 97.59)	0.59 (−2.17, 3.36)
**HbA1c**			
6 Months	5.44 (5.31, 5.57)	5.45 (5.37, 5.53)	0.01 (−0.15, 0.16)
12 Months	5.32 (5.17, 5.46)	5.28 (5.18, 5.37)	−0.04 (−0.21, 0.13)
**Self-report MVPA (mins.wk) †**			
6 Months	415.19 (294.31, 536.06)	469.48 (374.56, 564.40)	54.29 (−86.96, 195.54)
12 Months	415.53 (182.85, 648.21)	442.30 (329.19, 555.40)	26.77 (−225.90, 279.43)
**MVPA (mins.d) ‡** **,** **†**			
6 Months	67.35 (55.01, 79.70)	69.98 (61.54, 78.42)	2.63 (−12.22,17.48)
12 Months	66.76 (56.37, 77.15)	73.54 (61.56, 85.53)	6.78 (−9.19,22.76)
**Light Intensity Activity (mins.d) ‡** **,** **†**			
6 Months	224.57 (200.90, 248.24)	224.03 (206.60, 241.45)	−0.54 (−29.45, 28.37)
12 Months	219.20 (197.49, 240.92)	222.49 (197.50, 247.47)	3.28 (−30.64, 37.20)
**Daily Sitting Time (mins.d)**			
6 Months	682.86 (601.06, 764.66)	652.30 (602.49, 702.11)	−30.56 (−123.65, 62.53)
12 Months	568.56 (483.50, 653.61)	568.66 (518.92, 618.40)	0.10 (−100.25, 100.46)
**Sedentary Time (mins.d) ‡,†**			
6 Months	633.66 (592.52, 674.80)	661.33 (627.06, 695.60)	27.67 (−30.40, 85.74)
12 Months	649.40 (597.45, 701.35)	617.38 (576.14, 658.62)	−32.02 (−99.47, 35.43)
**Energy Intake (kj.d)**			
6 Months	9117.77 (8256.43, 9979.11)	8080.74 (7557.45, 8604.03)	−1037.03 (−2028.84, −45.22)
12 Months	9105.88 (8169.46, 10042.29)	8192.52 (7567.82, 8817.21)	−913.36 (−2030.75, 204.04)
**PSQI Global Score †**			
6 Months	6.76 (5.50, 8.02)	5.96 (5.24, 6.68)	−0.80 (−2.26, 0.67)
12 Months	5.96 (4.66, 7.26)	5.30 (4.35, 6.25)	−0.66 (−2.27, 0.95)
**Insomnia Severity Index**			
6 Months	8.03 (6.33, 9.74)	6.43 (5.41, 7.46)	−1.60 (−3.56, 0.36)
12 Months	8.16 (6.31, 10.00)	5.56 (4.33, 6.80)	−2.59 (−4.79, −0.39)
**Bed time Variability †**			
6 Months	2.78 (2.18, 3.39)	3.16 (2.76, 3.56)	0.38 (−0.35, 1.11)
12 Months	3.36 (2.65, 4.06)	3.02 (2.57, 3.46)	−0.34 (−1.18, 0.50)
**Wake time Variability ¥**			
6 Months	2.03 (1.56, 2.50)	2.44 (2.13, 2.75)	0.18 (−0.08, 0.44)
12 Months	2.49 (1.93, 3.04)	2.16 (1.82, 2.51)	−0.14 (−0.41, 0.13)
**Nightly Awakenings ‡**			
6 Months	2.28 (1.74, 2.83)	2.18 (1.85, 2.51)	−0.10 (−0.74, 0.53)
12 Months	1.97 (1.27, 2.67)	2.10 (1.65, 2.56)	0.13 (−0.71, 0.98)
**DASS-Depression †**			
6 Months	6.44 (4.88, 8.01)	4.96 (3.70, 6.23)	−1.48 (−3.54, 0.58)
12 Months	3.12 (1.32, 4.91)	4.84 (3.48, 6.20)	1.72 (−0.61, 4.05)
**DASS-Anxiety**			
6 Months	3.41 (2.10, 4.72)	3.19 (2.40, 3.99)	−0.21 (−1.71, 1.29)
12 Months	3.13 (1.75, 4.51)	2.68 (1.79, 3.58)	−0.44 (−2.07, 1.18)
**DASS-Stress**			
6 Months	9.96 (7.76, 12.17)	8.93 (7.60, 10.26)	−1.03 (−3.56, 1.50)
12 Months	9.34 (6.95, 11.73)	8.20 (6.61, 9.79)	−1.14 (−3.98, 1.71)

Notes: ‡ Measured using accelerometer. † Model included robust estimator. ¥ Modelled using Generalized Linear Mixed Model (Gaussian Log link). Group difference is the difference between groups (adjusted for baseline value of outcome) at each assessment.

**Table 3:** Baseline adjusted group differences between Enhanced and Traditional groups at 6 months and 12 months.

Outcome	Traditional	Enhanced	Group Difference (95% CI)
	M (95% CI)	M (95% CI)	
**Weight (kg)**			
6 Months	85.37 (83.71, 87.04)	87.28 (85.46, 89.10)	1.91 (−0.50, 4.31)
12 Months	86.34 (84.54, 88.14)	87.19 (85.06, 89.32)	0.85 (−1.89, 3.59)
**Waist Circ. (cm)**			
6 Months	95.12 (93.41, 96.84)	97.81 (95.93, 99.69)	2.69 (0.20, 5.18)
12 Months	95.42 (93.57, 97.27)	96.17 (93.96, 98.39)	0.75 (−2.08, 3.59)
**HbA1c**			
6 Months	5.46 (5.35, 5.57)	5.45 (5.33, 5.57)	−0.01 (−0.17, 0.15)
12 Months	5.36 (5.23, 5.48)	5.18 (5.02, 5.33)	−0.18 (−0.38, 0.02)
**Self-report MVPA (mins.wk) †**			
6 Months	555.61 (433.42, 677.79)	404.16 (260.54, 547.78)	−151.45 (−330.31, 27.41)
12 Months	532.45 (380.27, 684.63)	365.56 (219.20, 511.91)	−166.90 (−374.08, 40.29)
**MVPA (mins.d) ‡,†**			
6 Months	76.28 (60.97, 91.58)	61.54 (50.82, 72.26)	−14.74 (−37.03, 7.56)
12 Months	76.66 (58.12, 95.19)	70.43 (51.69, 89.18)	−6.22 (−35.66, 23.22)
**Light Intensity Activity (mins.d) ‡,†**			
6 Months	224.97 (197.83, 252.10)	209.09 (184.21, 233.96)	−15.88 (−56.55, 24.79)
12 Months	222.45 (184.93, 259.97)	208.14 (175.34, 240.95)	−14.31 (−68.62, 40.00)
**Daily Sitting Time (mins.d)**			
6 Months	677.99 (615.46, 740.53)	652.09 (583.53, 720.65)	−25.90 (−116.44, 64.64)
12 Months	549.21 (487.68, 610.74)	618.45 (530.56, 706.35)	69.24 (−39.00, 177.48)
**Sedentary Time (mins.d) ‡,†**			
6 Months	648.89 (612.76, 685.01)	688.27 (635.49, 741.06)	39.38 (−21.99, 100.76)
12 Months	594.00 (549.77, 638.24)	699.66 (635.44, 763.87)	105.66 (30.83, 180.48)
**Energy Intake (kj.d)**			
6 Months	8189.80 (7487.54, 8892.07)	8305.46 (7520.13, 9090.79)	115.65 (−939.39, 1170.70)
12 Months	8603.07 (7798.02, 9408.12)	7878.94 (6838.57, 8919.31)	−724.13 (−2053.96, 605.70)
**PSQI Global Score †**			
6 Months	6.14 (5.22, 7.06)	5.78 (4.62, 6.94)	−0.36 (−1.86, 1.14)
12 Months	5.34 (4.00, 6.67)	5.31 (4.13, 6.49)	−0.03 (−1.79, 1.74)
**Insomnia Severity Index**			
6 Months	6.27 (4.89, 7.66)	6.89 (5.33, 8.45)	0.62 (−1.44, 2.67)
12 Months	5.86 (4.31, 7.40)	5.19 (3.26, 7.13)	−0.66 (−3.12, 1.80)
**Bed Time Variability**			
6 Months	3.43 (2.79, 4.06)	2.83 (2.41, 3.25)	−0.60 (−1.37, 0.18)
12 Months	3.45 (2.80, 4.11)	2.38 (1.97, 2.78)	−1.08 (−1.86,−0.29)
**Wake time Variability ¥**			
6 Months	2.49 (2.07, 2.91)	2.39 (1.92, 2.85)	−0.04 (−0.29, 0.21)
12 Months	2.22 (1.80, 2.64)	2.09 (1.54, 2.64)	−0.06 (−0.38, 0.26)
**Nightly Awakenings ‡**			
6 Months	2.36 (1.91, 2.81)	2.38 (1.89, 2.86)	0.02 (−0.63, 0.67)
12 Months	2.48 (1.92, 3.04)	1.83 (1.03, 2.63)	−0.65 (−1.62, 0.32)
**DASS-Depression †**			
6 Months	4.61 (3.30, 5.92)	5.02 (2.74, 7.30)	0.41 (−2.25, 3.07)
12 Months	5.18 (3.47, 6.89)	3.82 (1.87, 5.77)	−1.36 (−4.04, 1.32)
**DASS-Anxiety**			
6 Months	3.35 (2.32, 4.39)	3.27 (2.11, 4.43)	−0.08 (−1.60, 1.43)
12 Months	3.40 (2.28, 4.52)	1.86 (0.49, 3.24)	−1.54 (−3.29, 0.21)
**DASS-Stress**			
6 Months	10.04 (8.29, 11.78)	8.02 (6.08, 9.96)	−2.02 (−4.58, 0.54)
12 Months	9.31 (7.30, 11.32)	7.10 (4.51, 9.70)	−2.21 (−5.48, 1.05)

Notes: ‡ Measured using accelerometer. † Model included robust estimator. ¥ Modelled using Generalized Linear Mixed Model (Gaussian Log link). Group difference is the difference between groups (adjusted for baseline value of outcome) at each assessment.

**Table 4:** Number of self-monitoring entries made by Move, Eat, Sleep participants using the Balanced app over 12 months.

Outcome	Traditional					Enhanced					Total					
	N	Mean	SD	Min	Max	N	Mean	SD	Min	Max	N	Mean	SD	Min	Max	*p*-Value
Total Entries †	41	156.5	102.8	0	342	39	140.4	83.3	1	353	80	148.7	93.6	0	353	0.440
Physical Activity Entries ‡	41	152.5	102.1	0	340	39	122.5	77.9	0	344	80	137.9	91.8	0	344	0.140
Food Entries	41	126.9	101.8	0	337	39	83.2	68.4	0	313	80	105.6	89.3	0	337	0.030
Sleep Entries	N/A	N/A	N/A	N/A	N/A	39	119.7	83.7	0	352	39	119.7	83.7	0	352	N/A
Body Weight Entries	41	62.2	81.9	0	327	39	38.2	59.5	0	349	80	50.5	72.4	0	349	0.140
Separate Physical Activity Behaviours																
Fitbit MVPA Entries	41	151.0	100.9	0	339	39	121.5	78.0	0	344	80	136.6	91.1	0	344	0.150
Daily Steps Entries	41	127.0	93.9	0	333	39	77.1	62.5	0	283	80	102.7	83.5	0	333	<0.001
Resistance Training Entries	41	127.2	96.5	0	331	39	68.5	62.4	0	274	80	98.6	86.4	0	331	<0.001

Notes: † Total entries is the number of self-monitoring entries for physical activity, diet, or weight in the Traditional group, and the number of self-monitoring entries for physical activity, diet, sleep or weight in the Enhanced group. ‡ Physical activity entries include Fitbit MVPA, Daily Steps, and Resistance Training entries.

## Supplementary Materials

The following are available online at https://www.mdpi.com/1660-4601/17/17/6200/s1. Figure S1: Individual weight change from baseline to 12 months by study group, Table S1: Overview of the intervention components and delivery methods in the Move, Eat & Sleep Study, Table S2: Comparison of completers and non-completers in Move, Eat & Sleep Study, Table S3: Weight, cardiovascular risk factors, lifestyle behaviours, and mental health characteristics at baseline, 6 month and 12 month health by Control & Pooled Intervention Groups, Table S4: Weight, cardiovascular risk factors, lifestyle behaviours, and mental health characteristics at baseline, 6 month and 12 month health Traditional & Enhanced Groups, Table S5: Sensitivity analyses using multiply imputed data (10 imputations); Baseline adjusted group differences between Pooled and Control Groups at 6 months and 12 months, Table S6: Sensitivity analyses using multiply imputed data (10 imputations). Baseline adjusted group differences between Enhanced and Traditional Groups at 6 months and 12 months, Table S7: System Usability Scores for Move, Eat & Sleep participants using the Balanced App at 6 months.
