# Supplementary material for: Efficacy of a Multi-component m-Health Weight-loss Intervention in Overweight and Obese Adults: A Randomised Controlled Trial
Source: Int J Environ Res Public Health. 2020 Aug 26;17(17):6200. doi: 10.3390/ijerph17176200 (PMC7503928; doi:10.3390/ijerph17176200)
Supplement: Supplementary file 1 [file ijerph-17-06200-s001.zip › MES main outcomes supp figures submission copy R1 140820.docx]

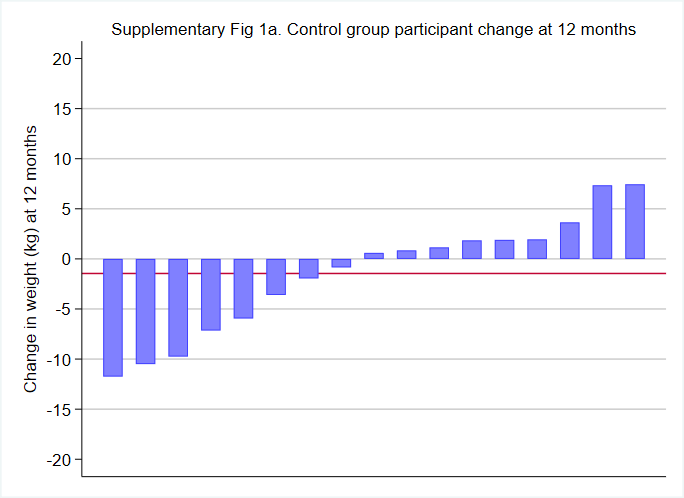


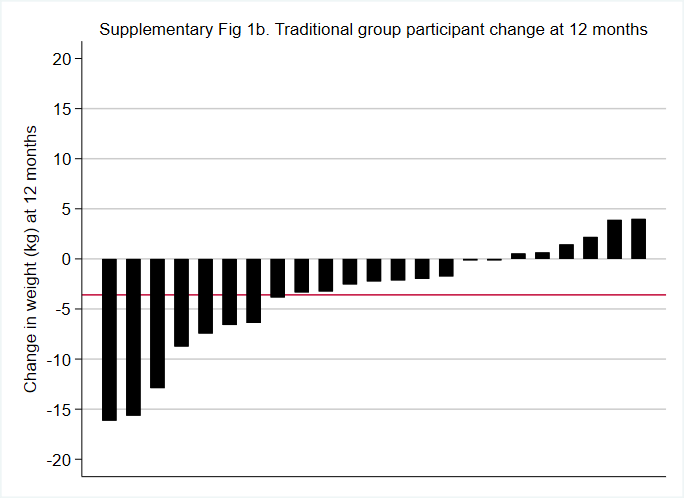


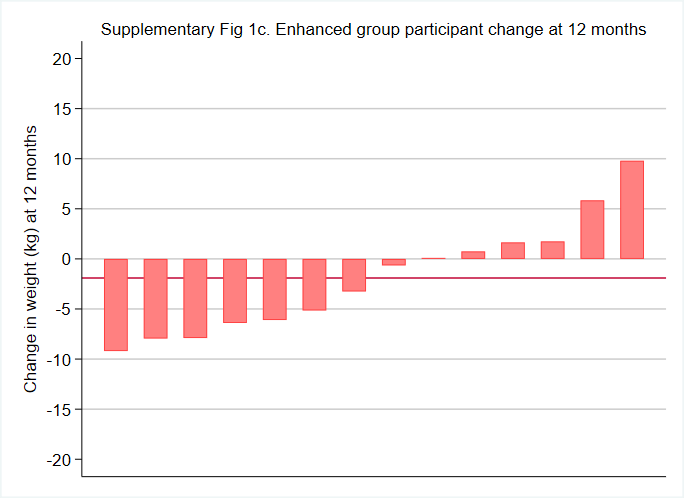


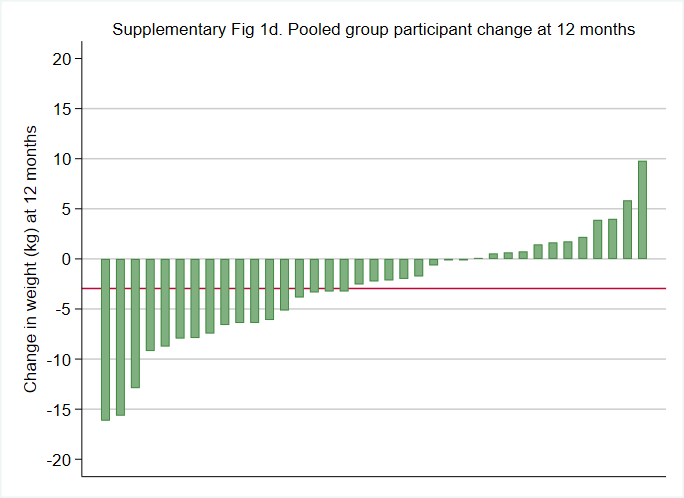


Supplementary Figure 1. Individual weight change from baseline to 12 months by study group

Notes. Only includes participants with weight measured at 12-months. Average (SD) weight loss at 12 months: Control (n=17) = -1.46 (5.85); Traditional (n=23)=-3.59 (5.60); Enhanced (n=14) = -1.91 (5.63); Pooled (n=37) = -2.95 (5.60).
