# Supplementary material for: Efficacy of a Multi-component m-Health Weight-loss Intervention in Overweight and Obese Adults: A Randomised Controlled Trial
Source: Int J Environ Res Public Health. 2020 Aug 26;17(17):6200. doi: 10.3390/ijerph17176200 (PMC7503928; doi:10.3390/ijerph17176200)
Supplement: Supplementary file 1 [file ijerph-17-06200-s001.zip › MES main outcomes supp materials submission copy R1 140820 mjd proof.docx]

**Supplementary material**

Supplementary Table S1. Overview of the intervention components and delivery methods in the Move, Eat & Sleep Study

| **Traditional group** | |  |  |  |  | | | | | | |
| --- | --- | --- | --- | --- | --- | --- | --- | --- | --- | --- | --- |
| **Behaviour change technique** | **Intervention component** | | **Delivery method** | **Frequency** | |  | **Study months**  **1-3 4-6 7-12** | | | | |
| Education | Physical activity: Accessed information related to the health benefits of physical activity, national physical activity guidelines, amount of physical activity required for weight loss, importance of physical activity to minimise sedentary behaviour, instructions for resistance training activities, and information on strategies and examples to create goals and action plans and overcome barriers to participation in physical activity.  Diet: Accessed information related to the health benefits of good dietary behaviours, national dietary guidelines, diet quality, energy deficit, core food/energy-dense food intake, fat intake, alcohol intake, emotional eating, healthy meals/snacks, portions sizes, interpreting food labels, and information on strategies and examples to create goals and action plans for healthy eating and monitoring energy intake. | | Participant handbook  Balanced app  SMS  Participant handbook  Balanced app  SMS | Ongoing  Ongoing  Weekly  Ongoing  Ongoing  Weekly | |  | ✓  ✓  ✓  ✓  ✓  ✓ | ✓  ✓  ✓  ✓  ✓  ✓ | | ✓  ✓  ✓  ✓ |  |
| Goal setting | Physical activity: Created goals for daily minutes of MVPA, daily step count, number of days per week of RT.  Diet: Created goals for daily intake of core foods, water, and energy-dense foods and drinks. | | Balanced app  Balanced app | Ongoing  Ongoing | |  | ✓  ✓ | ✓  ✓ | ✓  ✓ | |  |
| Action planning | Physical activity: Used an action planning tool to plan engagement in MVPA, RT, and increase in step count.  Diet: Used an action planning tool to plan healthy meals. | | Participant handbook  Participant handbook | Ongoing  Ongoing | |  | ✓  ✓ | ✓  ✓ | ✓  ✓ | |  |
| Self-monitoring | Physical activity: Manually logged daily step count and number of days per week of RT. Provided with a Fitbit Alta to assist with self-monitoring of daily minutes of MVPA.  Received self-monitoring prompt if non-usage of App occurred.^1^  Diet: Manually logged intake of foods related to food goals.  Manually logged food intake to self-monitor total energy intake.  Completed online quiz to self-monitor overall diet quality.  Received self-monitoring prompt if non-usage of App occurred.^1^  Weight: Manually logged weight. | | Balanced app  Fitbit Alta  SMS/Email  Balanced app  External platform^2^  External platform^3^  SMS/Email  Balanced app  Body weight scales | Daily  Weekly  Daily  4 days/week  Monthly  Weekly  Weekly | |  | ✓  ✓  ✓  ✓  ✓  ✓  ✓ | ✓  ✓  ✓  ✓  ✓  ✓  ✓ | ✓  ✓  ✓  ✓  ✓ | |  |
| Feedback | Physical activity: Received immediate feedback via App dashboard regarding daily minutes of MVPA using a traffic light system,^4^ and feedback about progress in relation to goals for MVPA, step count, and RT over four time points; one day, one week, three months, and all, using bar graphs.  Received feedback in relation to average weekly performance of MVPA, steps, and RT compared to personal goals.  Diet: Received feedback in relation to current dietary intake of core and energy-dense foods, macronutrient intake, micronutrient intake, and diet quality, compared with dietary recommendations.  Received immediate feedback via App dashboard regarding intake of foods compared with food goals using a traffic light system, and feedback about progress in relation to goals for food intake over four time points; one day, one week, three months, and all, using bar graphs.  Received feedback in relation to average weekly intake of core and energy-dense food intake, compared to personal goals.  Received immediate feedback in relation to total energy intake compared to personal energy intake target.  Received feedback in relation to overall diet quality compared to recommendations.  Weight: Received immediate feedback via App dashboard regarding weight using a traffic light system, and feedback about progress in relation to weight goal over four time points; one day, one week, three months, and all, using bar graphs.  Received feedback in relation to weight compared to personal goal. | | Balanced app  Email  In-person by dietitian  Balanced app  Email  External platform^2^  External platform^3^  Balanced app  Email | Daily  Weekly  Once^5^  Daily  Weekly  4 days/week  Monthly  Weekly  Weekly | |  | ✓  ✓  ✓  ✓  ✓  ✓  ✓  ✓  ✓ | ✓  ✓  ✓  ✓  ✓  ✓  ✓  ✓ | ✓  ✓  ✓  ✓  ✓ | |  |
|  |  | |  |  | |  |  |  |  | |  |
| **Enhanced group** | **Additional intervention components^6^** | |  |  | |  |  |  |  | |  |
|  |  | |  |  | |  | | |  | |  |
|  |  | |  |  | |  |  |  |  | |  |
| Education | Sleep: Accessed information related to the health benefits of good sleep health, national sleep duration guidelines, sleep hygiene practices (including stress management), and information on strategies and examples to create goals and action plans for healthier sleep. | | Participant handbook  Balanced app  SMS | Ongoing  Ongoing  Weekly | |  | ✓  ✓  ✓ | ✓  ✓  ✓ | ✓  ✓ | |  |
|  |  | |  |  | |  |  |  |  | |  |
| Goal setting | Sleep: Created goals for bed time and wake time, and number of sleep hygiene practices. | | Balanced app | Ongoing | |  | ✓ | ✓ | ✓ | |  |
|  |  | |  |  | |  |  |  |  | |  |
| Action planning | Sleep: Used an action planning tool to plan engagement in sleep-promoting behaviours. | | Balanced app | Ongoing | |  | ✓ | ✓ | ✓ | |  |
|  |  | |  |  | |  |  |  |  | |  |
| Self-monitoring  Feedback | Sleep: Manually logged sleep hygiene practices related to sleep hygiene goals. Provided with a Fitbit Alta to assist with self-monitoring of bed time and wake time, and sleep quality.  Received self-monitoring prompt if non-usage of App occurred.^1^  Sleep: Received immediate feedback via App dashboard regarding nightly sleep duration using a traffic light system, and feedback about progress in relation to goals for bed/wake time, sleep duration, sleep quality, and sleep hygiene over four time points; one day, one week, three months, and all, using bar graphs.  Received feedback in relation to average weekly sleep duration, bed time, wake time, and number of sleep hygiene practices, compared to personal goals. | | Balanced app  Fitbit Alta  SMS/Email  Balanced app  Email | Daily  Weekly  Daily  Weekly | |  | ✓  ✓  ✓  ✓ | ✓  ✓  ✓  ✓ | ✓  ✓ | |  |
|  |  | |  |  | |  |  |  |  | |  |

**Notes.**

Abbreviations: MVPA, Moderate-to-vigorous intensity physical activity; RT, resistance training; SMS, short messaging service

1. Criteria for receiving SMS self-monitoring prompt for physical activity, diet and sleep: if non-usage occurred on at least 4/7 days. Separate reminders for weekly self-monitoring of weight were integrated into the Personalised Weekly Summary. Participants will receive an additional email reminder to self-monitor physical activity, diet and sleep behaviours if they have received non-usage reminders for three consecutive weeks.

2. The external platform is CalorieKing website or the ControlMyWeight™ by CalorieKing app.

3. The external platform is the Healthy Eating Quiz™ [www.healthyeatingquiz.com.au](http://www.healthyeatingquiz.com.au)

4. Traffic light system; a green light indicated entries within 20% or meeting or exceeding goal; an orange light indicated entries between 20% and 35% of goal; a red light indicated entries more than 35% below goal.

5. Personalised dietary feedback provided to participants by a dietitian, based on the results generated from their Australian Eating Survey completed at baseline.

6. The Enhanced group received the same intervention components as the Traditional group, plus the sleep health components

Supplementary Table S2. Comparison of completers and non-completers in Move, Eat & Sleep Study

|  | **Completer** | **Non-Completer** | **Total** | **p-value** |
| --- | --- | --- | --- | --- |
|  | N=80 | N=36 | N=116 |  |
| Age (years) | 44.2 (9.9) | 45.0 (11.5) | 44.5 (10.4) | 0.70 |
| Gender (male) | 26 (32.5%) | 8 (22.2%) | 34 (29.3%) | 0.26 |
| Years Education | 16.5 (2.6) | 16.1 (3.7) | 16.4 (3.0) | 0.45 |
| Marital Status |  |  |  | 0.21 |
| Married/Defacto | 66 (82.5%) | 26 (72.2%) | 92 (79.3%) |  |
| Not Married | 14 (17.5%) | 10 (27.8%) | 24 (20.7%) |  |
| Employment |  |  |  | 0.33 |
| Full time | 46 (57.5%) | 16 (44.4%) | 62 (53.4%) |  |
| Part time / casual | 26 (32.5%) | 13 (36.1%) | 39 (33.6%) |  |
| Retired | 4 (5.0%) | 2 (5.6%) | 6 (5.2%) |  |
| Other | 4 (5.0%) | 5 (13.9%) | 9 (7.8%) |  |
| Weight (kg) | 90.2 (14.0) | 91.7 (15.0) | 90.7 (14.3) | 0.59 |
| Waist Circ. (cm) | 99.1 (10.8) | 100.7 (11.7) | 99.6 (11.0) | 0.49 |
| HbA1c | 5.4 (0.3) | 5.5 (0.7) | 5.4 (0.5) | 0.44 |
| Self-reported MVPA (mins.wk) | 296.8 (279.8) | 348.3 (335.7) | 312.8 (297.8) | 0.39 |
| MVPA (mins.d)ǂ | 56.2 (30.8) | 50.3 (31.9) | 54.4 (31.1) | 0.38 |
| Light Intensity Activity (mins.d)ǂ | 196.1 (73.9) | 192.8 (73.6) | 195.1 (73.5) | 0.84 |
| Daily Sitting Time (mins.d) | 708.2 (193.0) | 666.6 (210.1) | 695.3 (198.5) | 0.30 |
| Sedentary Time (mins.d)ǂ | 606.7 (145.7) | 609.0 (136.2) | 607.4 (142.3) | 0.94 |
| Energy Intake (kj.d) | 9555.8 (3153.6) | 9965.6 (3153.5) | 9683.0 (3145.6) | 0.52 |
| PSQI Global Score | 6.9 (2.9) | 7.1 (3.3) | 7.0 (3.0) | 0.74 |
| Insomnia Severity Index | 8.6 (4.8) | 9.1 (5.2) | 8.7 (4.9) | 0.59 |
| Nightly Awakeningsǂ | 2.1 (1.4) | 2.7 (1.9) | 2.3 (1.6) | 0.12 |
| DASS-Depression | 5.5 (5.1) | 9.9 (9.1) | 6.9 (6.9) | <0.001 |
| DASS-Anxiety | 4.2 (4.5) | 5.6 (6.4) | 4.7 (5.2) | 0.18 |
| DASS-Stress | 10.0 (6.2) | 12.7 (6.9) | 10.8 (6.5) | 0.036 |

Notes. Completer is defined as completing the 6 month assessment. Non completer is defined as not completing the 6 month assessment.ǂ measured using accelerometer.

Supplementary Table S3. Weight, cardiovascular risk factors, lifestyle behaviours, and mental health characteristics at baseline, 6 month and 12 month health by Control & Pooled Intervention Groups

|  | **Baseline** | | | | **6 Month** | | | |  | **12 Months** | | | | |
| --- | --- | --- | --- | --- | --- | --- | --- | --- | --- | --- | --- | --- | --- | --- |
|  | **Control** | | **Pooled** | | **Control** | | **Pooled** | |  | **Control** | | **Pooled** | |  |
|  | **N** | **M (SD)** | **N** | **M (SD)** | **N** | **M (SD)** | **N** | **M (SD)** | **d (95% CI)†** | **N** | **M (SD)** | **N** | **M (SD)** | **d (95% CI)†** |
| **Weight (kg)** | 36 | 92.50 (16.09) | 80 | 89.83 (13.41) | 21 | 90.19 (16.24) | 59 | 86.96 (14.39) | 0.22 (-0.28, 0.72) | 17 | 88.63 (16.85) | 37 | 84.63 (14.31) | 0.26 (-0.31, 0.84) |
| **Waist Circ. (cm)** | 36 | 99.72 (11.67) | 80 | 99.56 (10.80) | 21 | 98.48 (11.87) | 59 | 97.44 (10.08) | 0.10 (-0.40, 0.60) | 17 | 95.79 (13.19) | 37 | 95.13 (9.88) | 0.06 (-0.51, 0.63) |
| **HbA1c** | 36 | 5.29 (0.27) | 80 | 5.47 (0.55) | 21 | 5.41 (0.28) | 59 | 5.48 (0.37) | -0.18 (-0.67, 0.32) | 17 | 5.29 (0.30) | 37 | 5.29 (0.35) | 0.01 (-0.56, 0.59) |
| **Self-reported MVPA (mins.wk)** | 36 | 238.06 (239.19) | 80 | 346.38 (316.28) | 21 | 342.38 (281.41) | 60 | 473.50 (366.17) | -0.38 (-0.88, 0.12) | 17 | 361.76 (523.36) | 37 | 441.08 (322.92) | -0.20 (-0.78, 0.38) |
| **MVPA (mins.d)ǂ** | 29 | 49.22 (24.21) | 76 | 56.45 (33.26) | 19 | 65.31 (39.36) | 52 | 66.64 (34.83) | -0.04 (-0.56, 0.49) | 13 | 63.76 (37.68) | 29 | 72.88 (57.31) | -0.17 (-0.83, 0.48) |
| **Light Intensity Activity (mins.d)ǂ** | 29 | 188.03 (65.98) | 76 | 197.82 (76.38) | 19 | 236.30 (71.15) | 52 | 216.82 (79.63) | 0.25 (-0.28, 0.78) | 13 | 222.33 (82.27) | 29 | 219.87 (99.75) | 0.03 (-0.63, 0.68) |
| **Daily Sitting Time (mins.d)** | 36 | 678.06 (183.63) | 80 | 703.00 (205.46) | 21 | 666.02 (207.83) | 60 | 658.25 (209.03) | 0.04 (-0.46, 0.53) | 17 | 550.21 (165.89) | 37 | 599.40 (180.22) | -0.28 (-0.86, 0.30) |
| **Sedentary Time (mins.d)ǂ** | 33 | 583.98 (180.72) | 75 | 617.72 (121.72) | 18 | 630.94 (86.38) | 51 | 663.75 (150.81) | -0.24 (-0.78, 0.30) | 10 | 622.62 (109.96) | 26 | 641.34 (109.93) | -0.17 (-0.90, 0.56) |
| **Energy Intake (kj.d)** | 36 | 9152.64 (2810.27) | 80 | 9921.64 (3274.04) | 21 | 8886.90 (2890.33) | 60 | 8295.72 (2293.21) | 0.24 (-0.26, 0.74) | 17 | 8710.06 (2671.57) | 37 | 8308.62 (3068.57) | 0.14 (-0.44, 0.71) |
| **PSQI Global Score** | 36 | 6.72 (3.05) | 80 | 7.13 (2.97) | 21 | 6.43 (3.83) | 60 | 5.93 (3.61) | 0.14 (-0.36, 0.63) | 17 | 6.00 (3.77) | 37 | 5.38 (3.45) | 0.17 (-0.40, 0.75) |
| **Insomnia Severity Index** | 36 | 7.61 (4.79) | 80 | 9.21 (4.92) | 21 | 7.38 (5.31) | 60 | 6.60 (5.15) | 0.15 (-0.35, 0.65) | 17 | 7.82 (6.00) | 37 | 5.30 (4.32) | 0.52 (-0.07, 1.10) |
| **Bed Time Variability** | 36 | 3.04 (1.39) | 80 | 3.36 (1.69) | 21 | 2.81 (1.42) | 60 | 3.23 (1.65) | -0.27 (-0.76, 0.23) | 17 | 3.18 (1.34) | 37 | 3.02 (1.49) | 0.11 (-0.46, 0.69) |
| **Wake Time Variability** | 36 | 2.44 (1.33) | 80 | 2.44 (1.35) | 21 | 2.17 (0.73) | 60 | 2.45 (1.39) | -0.23 (-0.72, 0.27) | 17 | 2.39 (1.41) | 37 | 2.16 (1.09) | 0.20 (-0.38, 0.77) |
| **Nightly Awakeningsǂ** | 33 | 1.63 (1.43) | 75 | 2.57 (1.57) | 18 | 1.85 (1.13) | 51 | 2.38 (1.72) | -0.33 (-0.87, 0.21) | 10 | 1.80 (1.27) | 26 | 2.12 (1.33) | -0.24 (-0.97, 0.49) |
| **DASS-Depression** | 36 | 8.39 (7.87) | 80 | 6.17 (6.31) | 21 | 6.95 (6.83) | 60 | 5.00 (6.30) | 0.30 (-0.20, 0.80) | 17 | 4.00 (4.74) | 37 | 4.54 (5.86) | -0.10 (-0.67, 0.48) |
| **DASS-Anxiety** | 36 | 5.06 (5.77) | 80 | 4.47 (4.88) | 21 | 3.24 (4.07) | 60 | 3.53 (4.14) | -0.07 (-0.57, 0.43) | 17 | 3.18 (3.47) | 37 | 2.76 (4.20) | 0.11 (-0.47, 0.68) |
| **DASS-Stress** | 36 | 10.89 (6.79) | 80 | 10.82 (6.42) | 21 | 9.43 (7.65) | 60 | 9.20 (6.22) | 0.03 (-0.46, 0.53) | 17 | 8.35 (6.68) | 37 | 8.54 (6.05) | -0.03 (-0.60, 0.54) |

Notes. All table values based on observed observations. †Between groups differences expressed as Cohen's d are presented without adjustment for baseline value.

Supplementary Table S4. Weight, cardiovascular risk factors, lifestyle behaviours, and mental health characteristics at baseline, 6 month and 12 month health Traditional & Enhanced Groups

|  | **Baseline** | | | | **6 Month** | | | |  | **12 Months** | | | | |
| --- | --- | --- | --- | --- | --- | --- | --- | --- | --- | --- | --- | --- | --- | --- |
|  | **Traditional** | | **Enhanced** | | **Traditional** | | **Enhanced** | |  | **Traditional** | | **Enhanced** | |  |
|  | **N** | **M (SD)** | **N** | **M (SD)** | **N** | **M (SD)** | **N** | **M (SD)** | **d (95% CI)†** | **N** | **M (SD)** | **N** | **M (SD)** | **d (95% CI)†** |
| **Weight (kg)** | 41 | 88.91 (13.81) | 39 | 90.80 (13.09) | 32 | 85.35 (15.79) | 27 | 88.87 (12.56) | -0.24 (-0.76, 0.27) | 23 | 82.63 (13.86) | 14 | 87.92 (14.95) | -0.37 (-1.04, 0.30) |
| **Waist Circ. (cm)** | 41 | 99.61 (8.99) | 39 | 99.50 (12.54) | 32 | 95.94 (9.97) | 27 | 99.22 (10.10) | -0.33 (-0.84, 0.19) | 23 | 94.68 (9.82) | 14 | 95.86 (10.30) | -0.12 (-0.78, 0.55) |
| **HbA1c** | 41 | 5.48 (0.67) | 39 | 5.46 (0.40) | 32 | 5.45 (0.37) | 27 | 5.51 (0.38) | -0.17 (-0.68, 0.34) | 23 | 5.33 (0.39) | 14 | 5.22 (0.28) | 0.31 (-0.36, 0.98) |
| **Self-reported MVPA (mins.wk)** | 41 | 350.98 (357.72) | 39 | 341.54 (270.60) | 32 | 550.94 (366.27) | 28 | 385.00 (351.76) | 0.46 (-0.05, 0.97) | 23 | 508.70 (344.18) | 14 | 330.00 (258.69) | 0.57 (-0.11, 1.24) |
| **MVPA (mins.d)ǂ** | 40 | 56.29 (33.00) | 36 | 56.63 (34.01) | 29 | 66.67 (27.64) | 23 | 66.61 (42.89) | 0.00 (-0.55, 0.55) | 17 | 81.40 (70.53) | 12 | 60.80 (29.21) | 0.36 (-0.39, 1.10) |
| **Light Intensity Activity (mins.d)ǂ** | 40 | 196.08 (79.09) | 36 | 199.75 (74.32) | 29 | 210.50 (68.24) | 23 | 224.78 (93.02) | -0.18 (-0.73, 0.37) | 17 | 231.71 (112.63) | 12 | 203.10 (79.70) | 0.28 (-0.46, 1.02) |
| **Daily Sitting Time (mins.d)** | 41 | 736.83 (212.22) | 39 | 667.44 (194.48) | 32 | 695.80 (225.03) | 28 | 615.33 (183.73) | 0.39 (-0.12, 0.90) | 23 | 603.98 (187.02) | 14 | 591.89 (175.08) | 0.07 (-0.60, 0.73) |
| **Sedentary Time (mins.d)ǂ** | 37 | 629.72 (94.82) | 38 | 606.03 (143.52) | 27 | 644.66 (97.34) | 24 | 685.23 (194.48) | -0.27 (-0.82, 0.28) | 17 | 605.01 (104.27) | 9 | 709.96 (89.00) | -1.06 (-1.91, -0.18) |
| **Energy Intake (kj.d)** | 41 | 10396.85 (2989.02) | 39 | 9422.05 (3518.61) | 32 | 8702.59 (2129.17) | 28 | 7830.71 (2421.99) | 0.38 (-0.13, 0.89) | 23 | 9065.04 (3062.51) | 14 | 7065.93 (2743.71) | 0.68 (-0.01, 1.36) |
| **PSQI Global Score** | 41 | 6.95 (3.14) | 39 | 7.31 (2.81) | 32 | 5.91 (3.48) | 28 | 5.96 (3.82) | -0.02 (-0.52, 0.49) | 23 | 5.65 (3.70) | 14 | 4.93 (3.08) | 0.21 (-0.46, 0.87) |
| **Insomnia Severity Index** | 41 | 8.51 (5.16) | 39 | 9.95 (4.59) | 32 | 5.88 (4.28) | 28 | 7.43 (5.97) | -0.30 (-0.81, 0.21) | 23 | 5.57 (4.25) | 14 | 4.86 (4.55) | 0.16 (-0.50, 0.83) |
| **Bed Time Variability** | 41 | 3.21 (1.59) | 39 | 3.52 (1.79) | 32 | 3.34 (1.89) | 28 | 3.11 (1.36) | 0.14 (-0.37, 0.65) | 23 | 3.36 (1.77) | 14 | 2.46 (0.56) | 0.62 (-0.06, 1.30) |
| **Wake Time Variability** | 41 | 2.55 (1.40) | 39 | 2.32 (1.30) | 32 | 2.56 (1.47) | 28 | 2.33 (1.32) | 0.17 (-0.34, 0.67) | 23 | 2.34 (1.29) | 14 | 1.87 (0.59) | 0.43 (-0.24, 1.10) |
| **Nightly Awakeningsǂ** | 37 | 2.32 (1.28) | 38 | 2.82 (1.79) | 27 | 2.42 (1.43) | 24 | 2.35 (2.03) | 0.04 (-0.51, 0.59) | 17 | 2.39 (1.26) | 9 | 1.60 (1.37) | 0.60 (-0.23, 1.42) |
| **DASS-Depression** | 41 | 6.54 (7.43) | 39 | 5.79 (4.94) | 32 | 4.63 (5.12) | 28 | 5.43 (7.50) | -0.13 (-0.63, 0.38) | 23 | 5.65 (6.84) | 14 | 2.71 (3.20) | 0.51 (-0.17, 1.18) |
| **DASS-Anxiety** | 41 | 4.73 (5.23) | 39 | 4.21 (4.54) | 32 | 3.38 (4.50) | 28 | 3.71 (3.76) | -0.08 (-0.59, 0.43) | 23 | 3.57 (5.04) | 14 | 1.43 (1.65) | 0.52 (-0.16, 1.19) |
| **DASS-Stress** | 41 | 10.83 (6.77) | 39 | 10.82 (6.12) | 32 | 9.88 (5.90) | 28 | 8.43 (6.59) | 0.23 (-0.28, 0.74) | 23 | 9.65 (6.89) | 14 | 6.71 (3.89) | 0.49 (-0.18, 1.16) |

Notes. All table values based on observed observations. †Between groups differences expressed as Cohen's d are presented without adjustment for baseline value.

Supplementary Table S5. Sensitivity analyses using multiply imputed data (10 imputations); Baseline adjusted group differences between Pooled and Control Groups at 6 months and 12 months

|  | **Control** | **Pooled Intervention** |  |
| --- | --- | --- | --- |
|  | **M (95%CI)** | **M (95%CI)** | **Group Difference (95%CI)** |
| **Weight (kg)** |  |  |  |
| 6 Months | 88.36 (83.97, 92.75) | 87.03 (83.79, 90.28) | -1.33 (-5.38, 2.72) |
| 12 Months | 89.18 (83.77, 94.58) | 86.57 (82.58, 90.55) | -2.61 (-8.42, 3.20) |
| **Waist Circ. (cm)** | |  |  |
| 6 Months | 95.44 (91.30, 99.58) | 93.67 (90.39, 96.95) | -1.77 (-5.56, 2.02) |
| 12 Months | 94.79 (90.20, 99.38) | 92.02 (88.02, 96.03) | -2.77 (-7.73, 2.19) |
| **HbA1c** |  |  |  |
| 6 Months | 5.37 ( 5.18, 5.56) | 5.42 ( 5.29, 5.54) | 0.05 (-0.13, 0.23) |
| 12 Months | 5.25 ( 5.07, 5.42) | 5.25 ( 5.10, 5.39) | 0.00 (-0.19, 0.19) |
| **Self-report MVPA (mins.wk)†** | |  |  |
| 6 Months | 482.68 (298.47, 666.89) | 514.21 (359.58, 668.84) | 31.53 (-153.37,216.43) |
| 12 Months | 489.82 (253.05, 726.58) | 489.47 (340.67, 638.27) | -0.35 (-242.51,241.81) |
| **MVPA (mins.d)ǂ†** | |  |  |
| 6 Months | 92.01 (14.11, 169.92) | 89.47 (13.76, 165.17) | -2.55 (-20.21,15.11) |
| 12 Months | NR | NR | NR |
| **Light Intensity Activity (mins.d)ǂ†** | |  |  |
| 6 Months | 231.29 (82.74, 379.84) | 215.54 (68.46, 362.62) | -15.75 (-48.68,17.17) |
| 12 Months | NR | NR | NR |
| **Daily Sitting Time (mins.d)** | |  |  |
| 6 Months | 651.30 (558.44, 744.16) | 652.69 (580.85, 724.54) | 1.39 (-105.18,107.97) |
| 12 Months | 579.02 (481.40, 676.63) | 600.88 (537.67, 664.09) | 21.86 (-74.84,118.57) |
| **Sedentary Time (mins.d)ǂ†** | |  |  |
| 6 Months | 871.28 (740.77, 1001.79) | 871.60 (729.41, 1013.79) | 0.32 (-49.94,50.58) |
| 12 Months | NR | NR | NR |
| **Energy Intake (kj.d)** | |  |  |
| 6 Months | 8826.54 (7547.70, 10105.38) | 8102.31 (7082.87, 9121.76) | -724.23 (-1857.53,409.08) |
| 12 Months | 9068.50 (7873.49, 10263.51) | 8076.21 (7203.51, 8948.91) | -992.29 (-2256.00,271.43) |
| **PSQI Global Score †** | |  |  |
| 6 Months | 6.76 ( 5.04, 8.47) | 6.54 ( 5.12, 7.95) | -0.22 (-1.96, 1.53) |
| 12 Months | 6.42 ( 4.50, 8.35) | 6.26 ( 4.78, 7.73) | -0.17 (-2.07, 1.74) |
| **Insomnia Severity Index** | |  |  |
| 6 Months | 7.77 ( 5.11, 10.43) | 7.17 ( 5.50, 8.83) | -0.60 (-3.21, 2.01) |
| 12 Months | 8.10 ( 5.72, 10.48) | 6.70 ( 4.81, 8.58) | -1.40 (-3.87, 1.07) |
| **Bed time Variability†** | | |  |
| 6 Months | 3.09 ( 2.19, 4.00) | 3.48 ( 2.68, 4.29) | 0.39 (-0.59, 1.37) |
| 12 Months | 3.54 ( 2.38, 4.70) | 3.48 ( 2.67, 4.29) | -0.06 (-1.47, 1.35) |
| **Wake time Variability†** | | |  |
| 6 Months | 2.31 ( 1.68, 2.94) | 2.68 ( 2.03, 3.33) | 0.37 (-0.19, 0.94) |
| 12 Months | 2.55 ( 1.61, 3.49) | 2.36 ( 1.74, 2.98) | -0.19 (-1.14, 0.77) |
| **Nightly Awakeningsǂ** | | |  |
| 6 Months | -0.37 (-2.12, 1.38) | -0.21 (-1.82, 1.40) | 0.16 (-0.61, 0.93) |
| 12 Months | NR | NR | NR |
| **DASS-Depression †** | | |  |
| 6 Months | 5.66 ( 2.73, 8.59) | 4.91 ( 3.06, 6.76) | -0.75 (-3.68, 2.17) |
| 12 Months | 4.34 ( 2.08, 6.60) | 4.75 ( 2.72, 6.78) | 0.41 (-2.44, 3.25) |
| **DASS-Anxiety** | | |  |
| 6 Months | 2.44 ( 0.73, 4.15) | 3.21 ( 1.95, 4.48) | 0.77 (-0.83, 2.37) |
| 12 Months | 2.57 ( 0.66, 4.49) | 2.50 ( 1.10, 3.90) | -0.07 (-1.83, 1.68) |
| **DASS-Stress** | | |  |
| 6 Months | 9.43 ( 6.41, 12.45) | 9.36 ( 7.33, 11.39) | -0.07 (-3.04, 2.90) |
| 12 Months | 8.73 ( 5.72, 11.74) | 8.72 ( 6.36, 11.09) | -0.00 (-2.92, 2.91) |

Notes:ǂ measured using accelerometer.† model included robust estimator.¥ modelled using Generalized Linear Mixed Model (Gaussian Log link). NR not reported as imputation model did not converge for these outcomes at specified time point.

Supplementary Table S6. Sensitivity analyses using multiply imputed data (10 imputations). Baseline adjusted group differences between Enhanced and Traditional Groups at 6 months and 12 months

|  | **Traditional** | **Enhanced** |  |
| --- | --- | --- | --- |
|  | **M (95%CI)** | **M (95%CI)** | **Contrast (95%CI)** |
| **Weight (kg)** |  |  |  |
| 6 Months | 86.93 (83.09, 90.77) | 87.04 (83.22, 90.86) | 0.11 (-3.90, 4.13) |
| 12 Months | 86.52 (81.95, 91.08) | 86.63 (81.44, 91.83) | -0.01 (-6.43, 6.42) |
| **Waist Circ. (cm)** | |  |  |
| 6 Months | 92.90 (88.83, 96.98) | 93.93 (89.59, 98.27) | 1.03 (-3.18, 5.23) |
| 12 Months | 91.43 (86.54, 96.32) | 92.45 (86.86, 98.04) | 0.68 (-5.62, 6.97) |
| **HbA1c** |  |  |  |
| 6 Months | 5.37 ( 5.23, 5.52) | 5.40 ( 5.21, 5.60) | 0.03 (-0.17, 0.23) |
| 12 Months | 5.22 ( 5.03, 5.41) | 5.25 ( 4.95, 5.55) | 0.00 (-0.21, 0.21) |
| **Self-report MVPA (mins.wk)†** | |  |  |
| 6 Months | 556.93 (391.17, 722.68) | 438.50 (217.48, 659.53) | -118.42 (-308.88,72.04) |
| 12 Months | 501.73 (297.86, 705.61) | 383.31 (92.33, 674.28) | -55.95 (-280.08,168.19) |
| **MVPA (mins.d)ǂ†** | |  |  |
| 6 Months | 80.80 (10.73, 150.87) | 81.53 ( 8.06, 155.00) | 0.73 (-16.67,18.14) |
| 12 Months | NR | NR | NR |
| **Light Intensity Activity (mins.d)ǂ†** | |  |  |
| 6 Months | 210.63 (46.00, 375.27) | 211.96 (44.21, 379.70) | 1.32 (-33.66,36.31) |
| 12 Months | NR | NR | NR |
| **Daily Sitting Time (mins.d)** | |  |  |
| 6 Months | 671.23 (572.13, 770.34) | 647.64 (555.60, 739.68) | -23.60 (-130.20,83.01) |
| 12 Months | 594.74 (516.82, 672.65) | 571.14 (444.95, 697.34) | 27.03 (-83.84,137.90) |
| **Sedentary Time (mins.d)ǂ†** | |  |  |
| 6 Months | 865.76 (725.87, 1005.65) | 883.97 (735.79, 1032.15) | 18.21 (-38.12,74.54) |
| 12 Months | NR | NR | NR |
| **Energy Intake (kj.d)** | |  |  |
| 6 Months | 8200.60 (6998.11, 9403.08) | 8040.32 (6642.58, 9438.06) | -160.28 (-1326.43,1005.88) |
| 12 Months | 8197.18 (7026.28, 9368.07) | 8036.90 (6312.73, 9761.07) | -206.80 (-1920.08,1506.49) |
| **PSQI Global Score†** | |  |  |
| 6 Months | 6.37 ( 4.86, 7.87) | 6.70 ( 4.99, 8.41) | 0.34 (-1.29, 1.96) |
| 12 Months | 6.23 ( 4.26, 8.19) | 6.56 ( 4.22, 8.91) | 0.04 (-2.25, 2.34) |
| **Insomnia Severity Index** | | |  |
| 6 Months | 6.82 ( 4.77, 8.87) | 7.50 ( 5.29, 9.72) | 0.68 (-1.74, 3.11) |
| 12 Months | 6.53 ( 4.14, 8.91) | 7.21 ( 4.36, 10.06) | 0.32 (-2.45, 3.10) |
| **Bed time Variability** | | |  |
| 6 Months | 3.67 ( 2.62, 4.73) | 3.34 ( 2.19, 4.49) | -0.33 (-1.63, 0.96) |
| 12 Months | 3.72 ( 2.70, 4.73) | 3.39 ( 1.88, 4.89) | -0.44 (-1.74, 0.87) |
| **Wake time Variability†** | | |  |
| 6 Months | 2.79 ( 1.92, 3.67) | 2.80 ( 2.02, 3.58) | 0.01 (-0.85, 0.87) |
| 12 Months | 2.48 ( 1.61, 3.35) | 2.49 ( 1.32, 3.66) | -0.01 (-0.95, 0.92) |
| **Nightly Awakeningsǂ** | | |  |
| 6 Months | -0.05 (-2.01, 1.91) | -0.36 (-2.16, 1.45) | -0.31 (-1.07, 0.45) |
| 12 Months | NR | NR | NR |
| **DASS-Depression†** | | |  |
| 6 Months | 4.84 ( 2.81, 6.87) | 5.31 ( 2.79, 7.84) | 0.47 (-2.38, 3.32) |
| 12 Months | 5.19 ( 2.96, 7.41) | 5.66 ( 2.45, 8.87) | -0.56 (-3.48, 2.35) |
| **DASS-Anxiety** | | |  |
| 6 Months | 3.29 ( 1.65, 4.93) | 3.55 ( 1.90, 5.20) | 0.26 (-1.43, 1.94) |
| 12 Months | 2.81 ( 1.03, 4.59) | 3.07 ( 0.86, 5.28) | -0.21 (-2.26, 1.85) |
| **DASS-Stress** | | |  |
| 6 Months | 9.83 ( 7.22, 12.43) | 9.07 ( 6.14, 11.99) | -0.76 (-4.25, 2.73) |
| 12 Months | 9.03 ( 6.27, 11.78) | 8.27 ( 4.07, 12.46) | -0.42 (-4.33, 3.49) |

Notes: ǂ measured using accelerometer.† model included robust estimator.¥ modelled using Generalized Linear Mixed Model (Gaussian Log link).

Supplementary Table S7. System Usability Scores for Move, Eat & Sleep participants using the Balanced App at 6 months

|  | Traditional | Enhanced | Total |
| --- | --- | --- | --- |
|  | N=32 | N=28 | N=60 |
| Like to use Balanced Frequently | 2.3 (1.2) | 2.1 (1.2) | 2.3 (1.2) |
| Balanced was easy to use | 3.3 (0.5) | 2.9 (1.0) | 3.1 (0.8) |
| Various functions of Balanced well integrated | 2.7 (0.9) | 2.4 (1.2) | 2.6 (1.0) |
| Most people could learn to use Balanced quickly | 3.3 (0.6) | 2.9 (1.1) | 3.2 (0.9) |
| I felt confident using Balanced | 3.1 (0.8) | 2.7 (1.1) | 2.9 (1.0) |
| Found Balanced unnecessarily complex | 3.2 (0.8) | 2.6 (1.1) | 2.9 (1.0) |
| Balanced needed support of a technical person to use | 3.6 (0.5) | 3.4 (0.9) | 3.5 (0.7) |
| Too much inconsistency in Balanced | 2.8 (0.8) | 2.6 (1.0) | 2.7 (0.9) |
| The Balanced app very cumbersome to use | 2.9 (1.0) | 2.6 (1.2) | 2.8 (1.1) |
| I needed to learn a lot of things before using Balanced | 3.2 (0.7) | 3.0 (1.1) | 3.1 (0.9) |
| Overall SUS Score | 76.2 (13.6) | 67.4 (22.6) | 72.2 (18.6) |

Note. No difference between overall SUS Scores between groups (t(93)=1.617, p = 0.109. SUS Scores: 1 = Strongly disagree; 5 = Strongly Agree.
